# Linguistics and semiotics in indigenous and non-indigenous people’s training and performance in nursing

**DOI:** 10.1590/1980-220X-REEUSP-2025-0023en

**Published:** 2025-08-04

**Authors:** Nádile Juliane Costa de Castro, Purupramare Lima Gavião, Antônio Luis Parlandin dos Santos, Fernanda Teixeira Paes

**Affiliations:** 1 Universidade Federal do Pará, Programa de Pós-Graduação em Enfermagem, Belém, PA, Brazil.; 2 Ministério da Saúde, Secretaria de Saúde Indígena, Distrito Guamá. Tocantins, Belém, PA, Brazil.; 3 Universidade Federal do Pará, Faculdade de Letras. Belém, PA, Brazil.

**Keywords:** Learning, Nurses, Indigenous Peoples, Staff Development, Diversity, Equity, Inclusion

## Abstract

**Objective:**

To reflect on linguistics’ and semiotics’ contributions to intercultural training and indigenous and non-indigenous nurses’ work in the Brazilian Health System, highlighting the challenges, potential and strategies of inclusive communication in healthcare of indi­genous peoples.

**Method:**

This is a study with a theoretical-reflexive approach, supported by contemporary literature, as well as by the experience and identity of its authors, in order to elucidate the contributions brought by those sciences, in light of critical interculturality.

**Results:**

It was organized into two cores, following the sequence of ideas on training, implementation and action: Linguistics and semiotics in intercultural training: strategies for equity in professional practice; and Language translation and interpretation: paths to equity in healthcare.

**Final considerations:**

The inclusion of specific linguistic and semiotic content in undergraduate courses, associated with the development of bilingual tools and the adoption of policies that respect the diverse indigenous worldviews, reinforces commitments to equity and social justice in healthcare services provided by the Brazilian public health system.

## INTRODUCTION

The implementation of health and education policies involves inclusive processes^(1)^, such as dialogue between linguistic and cultural diversity in educational spaces and healthcare services^(2)^. These processes are similar to discussions on interculturality, contributing with theoretical and practical insights into the training and health activities of indigenous and non-indigenous nurses. This fact is therefore in line with sustainable, inclusive and equitable learning environments^(1,3)^, based on the Brazilian Health System (In Portuguese, *Sistema Único de Saúde* – SUS)^(4,5)^ principles and the Sustainable Development Goals (SDGs), especially SDGs 3 and 4 (Good health and well-being and Quality education), respectively, providing critical, reflective and civic actions to educational institutions.

This movement contributes to the expansion of the body of knowledge and promotes ruptures with Eurocentric epistemologies, a central paradigm in current training, converging towards the inclusion of epistemologies from the Global South aligned with indigenous medicines and care practices of the 305 indigenous peoples present in Brazilian territory^(3)^. Likewise, these changes provide theoretical support for the problematization of ethnic peculiarities and health needs of territories^(6)^, within the perspectives of concepts on health and intercultural education^(4,5)^. Furthermore, they collaborate to face emerging issues, due to their multi-epistemic movements, capable of identifying new pedagogical nuances and undertaking dialogicity with those usually applied.

Within SUS, this plural scenario is identified mainly in the Indigenous Healthcare Subsystem (In Portuguese, *Subsistema de Atenção à Saúde Indígena* SASISUS), which guides healthcare of indigenous peoples^(7)^, which demands a political approach that addresses inclusion and interdisciplinarity^(2,3)^. In this regard, language sciences can contribute, through linguistics and semiotics^(8)^, in order to bring concepts about the forms of communication of ethnic groups assisted by SASISUS, aiming to understand aggregated symbols and identify meanings attributed to elements in the cultures in question^(9)^.

Nursing, as a leading figure in this scenario, demands advances in diversity and communication, which are important for safe action and good practices in healthcare^(4)^, which do not result, consequently, in inaccurate diagnoses, inadequate treatments and unfavorable health outcomes^(7)^. Therefore, this study is justified by its proposals to fill knowledge gaps, referring to the underutilization of language sciences in communications with indigenous peoples, and to compose a response to the Brazilian National Policy for Healthcare for Indigenous Peoples.

Furthermore, the current state is fundamental in its epistemological alignment with indigenous authors, who recognize the ethnic, cultural and worldview pluralities of their societies. This discussion also demonstrates the inseparability between training for working in indigenous territories and other areas of knowledge, such as linguistics, which mediate communication between users and Multidisciplinary Indigenous Health Teams (MIHT), from Primary Healthcare (PHC)^(7)^, where nurses, physicians, indigenous health agents, among others, are found.

Studies in this regard highlight the challenges to communication between MIHT and indigenous patients; challenges that occur inside and outside the territories, in PHC and at other levels of healthcare, associated mainly with undergraduate training. On the other hand, such opportunities can bring excellent contributions, due to indigenous people’s access to higher education, based on Law 14,723/2023 (Quota Law), which allows the training of indigenous nurses.

However, considering the relationships between indigenous and non-indigenous students, this access must have pedagogical support, linguistic and semiotic interpretations, and inclusive communications, systemic understandings that are still little explored^(7,10)^. This aspect highlights other training issues, such as overcoming intolerance and linguistic prejudice, which demands epistemological changes, in order to enable interventions in social practice in health, aiming to achieve differentiated care^(11,12,13)^.

These interventions promote equity in the mediation of healthcare services, shape new structures and infrastructures^(4)^ within healthcare services, and promote research that enriches the understanding of training needs for work in SASISUS^(4,11)^, contributing to reducing social and health inequities, considering the promotion of inclusive and socially engaged qualifications^(1)^.

Thus, the question was raised about the best strategies to be used to qualify learning processes in the training of indigenous and non-indigenous nurses, as well as in their work with indigenous users, with the purpose of pointing out limitations and possibilities for implementing learning and care processes. In addition to these, there are constructs that can be developed transversally, through extension and research, and the intervention strategies that can/should be undertaken to promote effective healthcare practices for indigenous peoples. Finally, this study aims to reflect on linguistics’ and semiotics’ contributions to indigenous nurses’ intercultural training and work in SUS, highlighting the challenges, potentialities and strategies of inclusive communication in healthcare for indigenous peoples.

Linguistics and semiotics provide essential tools for understanding communication processes, especially in intercultural and multilingual contexts, such as the training of nurses who work with indigenous peoples. These fields enable critical and reflective approaches to the ways in which language and symbols shape healthcare relationships, directly contributing to the training of culturally competent professionals. In this study, reflections will be conducted based on dialogue with authors from the Global South, observing studies on intercultural health, which have guided discussions on indigenous healthcare, in order to enable dialogicity between the identities of indigenous authors and academic training.

### Conceptual Aspects of Linguistics and Semiotics and Indigenous Perspectives

Linguistics is the science that studies verbal language in its multiple dimensions, contemplating descriptive, historical and theoretical aspects of language, as a structured system of communication, and its constant elaboration and re-elaboration^(8)^. This differs from semiotics, which is dedicated to the study of signs, encompassing systems and processes represented by symbols and clues^(14)^, in their forms, types and effects. While linguistics focuses on structures and functioning of languages, in their deictic-referential, logical, pragmatic-discursive, syntactic and semantic dimensions^(15)^, semiotics investigates how meanings are constructed, shared and interpreted, through the different systems of signs present in different cultures^(14)^.

In this sense, recognizing the subareas of linguistics, such as semantics, pragmatics and sociolinguistics, makes it possible to understand meanings, uses of language in certain contexts and social relations, respectively^(8)^. Furthermore, distinguishing cultural semiotics’ function is fundamental to the study of signs in indigenous contexts, due to the multimodality (verbal, visual, gestural, spatial) identified in them and the incorporation of different elements into the analysis of symbolic practices of different ethnic groups^(16,17)^. This separation is fundamental to understanding how fields of knowledge contribute differently to intercultural health education, especially in multilingual contexts^(9)^.

On the other hand, it is important to emphasize that linguistics and semiotics have their conceptual and methodological bases built on Western epistemology^(8,14)^. In this regard, there are risks in applying the Eurocentric concepts that characterize them to indigenous realities without due problematization, since the Brazilian territory’s indigenous peoples have their own systems of production and interpretation of meanings, intrinsically connected to their worldviews^(16)^. Therefore, it is necessary to observe an “indigenous semiotics” that recognizes their own systems of signs and meanings, which are manifested through body paintings, graphics, songs, narratives, rituals and their own relationships with the territory^(17)^.

## METHOD

This is a theoretical-reflective study, supported by scientific literature on linguistics and semiotics, referring to the training of indigenous and non-indigenous nurses, as well as the perceptions, experiences and identities of its authors, in order to elucidate the relationships established between the instruction and performance of these professionals. This research was carried out between November 2023 and December 2024, and was based on the so-called intercultural education, by Gersen Baniwa^(16)^, through which the author states that it is necessary to take a critical look at epistemologies that have been applied in formative processes, being essential to break this hegemony and conduct dialogues with other ways of thinking.

Linguistic and cultural qualifications are central and support improvements in the communicational effectiveness of interactions between nurses and indigenous patients^(18)^, an aspect that, in light of thought and language, transcends the understanding of social phenomena. In the training of these workers, such improvements have the potential for the development of complex intercultural actions^(2)^, both at the social level—initially—and at the individual level—later on^(14)^ —, which are decisive for understanding and improving communication and learning in multicultural and multilingual health contexts^(8,9)^ of action.

In order for nursing training to be inclusive, it is argued that it should be mediated by indigenous concepts from linguistics and semiotics^(17)^, which should contribute to the promotion of more comprehensive communicative practices, the reduction of linguistic and cultural challenges within SUS, and the strengthening of public policies oriented towards equity. This paradigm can also support more holistic learning in nursing^(19)^ and targets present in SDGs 3 and 4, notably the development and training of healthcare professionals (item 3c), and the appreciation of cultural diversity (item 4.7)^(19,20)^.

This text is structured into two thematic areas: Linguistics and semiotics in intercultural training: strategies for equity in professional practice; and Translation and interpretation of languages: paths towards equity in healthcare. This is a theoretical-reflective work, and there is no need for assessment by the CEP/CONEP system.

### Development

As a starting point, it is necessary to understand language as a technical instrument and as a means of building intercultural relationships, mediated by symbols, values ​​and specific contexts of indigenous communities. In this sense, based on the authors’ experiences, it is possible to identify challenges and potentialities to point out pragmatic strategies.

**Figure 1:**
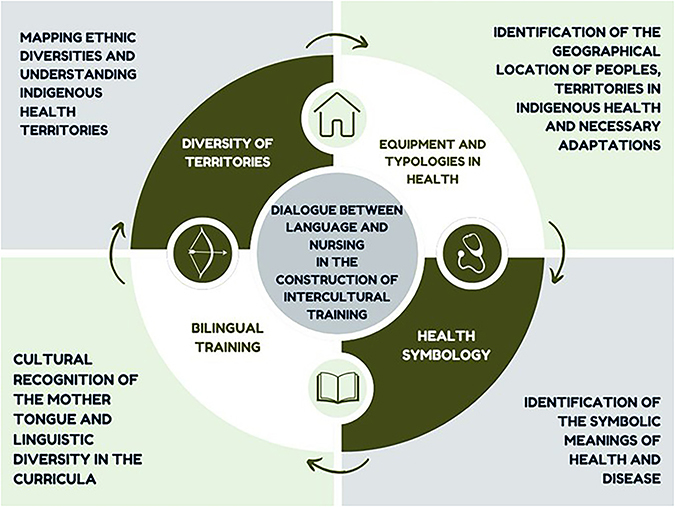
Representation of dialogicity in the intercultural training of nurses.

Source: authors, 2025.

Based on the proposed dialogicity, it is understood that social interaction is central to the development of thought and language^(16)^, directly reflecting on the training of healthcare professionals. On the other hand, the issue involves other aspects, such as the identification of ethnic diversity^(16)^, indigenous territories and health equipment and their typologies (Figure 1) so that indigenous and non-indigenous professionals receive an education that values their mother tongues and understands the symbolic meanings attributed to health and disease by each group—and the implications of these in care processes.

### Linguistics and Semiotics in Intercultural Training: Strategies for Equity in Professional Practice

Intercultural competence^(10,15)^ and recognition of multilingualism are crucial for nurses, especially those working in SASISUS, in regions with different indigenous peoples. These aspects are fundamental to the acquisition of equitable healthcare^(4,5)^, whose abilities to identify and navigate multiple languages broaden the understanding of cultural nuances^(4)^, which are intrinsic to communication, and the interpretation of health messages^(13)^. Furthermore, establishing culturally sensitive communication enriches patient-nurse interaction^(5)^ and also promotes a more holistic approach in terms of care^(13)^.

Improved communication encompasses the ability to understand and be understood in the multilingual^(18)^ and multicultural spectrums, which are intrinsic to the effectiveness and quality of care provided within SUS, which transcends the simple transmission of information. Likewise, this improvement emerges as a fundamental pillar for the appreciation of cultural diversity, recognizing that each individual brings with them knowledge and practices that shape their perception of health, as pointed out in the SDGs^(19,20)^. When well structured, such improvement can equip nurses with the tools to adequately transfer critical health knowledge^(5)^, build relationships of trust and empathy, and correctly systematize the care needed for so- called humanized care, especially in SASISUS^(10,13)^.

Furthermore, the ability to communicate and understand cultural nuances increases accessibility and inclusion in healthcare^(2,13)^, facilitating the implementation of health promotion and disease prevention strategies that respect the singularities of each ethnic group^(13)^. Thus, at the interface between linguistics, semiotics and equity^(8,9)^, it is pointed out that the inclusion of indigenous people at different levels of education, such as undergraduate and graduate studies^(1,3)^, is a bridge to professional development^(19,20)^ and to the strengthening of cultural and epistemic representations in educational spaces.

Furthermore, such improvement can promote the construction of more humanized and respectful care practices, aligned with indigenous specificities and worldviews, in addition to contributing to the reduction of health inequities and the consolidation of critical and socially fair training in SUS. To this end, it is necessary to insert these paradigms into educational processes and recognize the fundamental role of linguistics in conducting the process, since it is based on resources that analyze its structural relations^(5)^, covering the semantic, deictic-referential and pragmatic-discursive dimensions^(8,9,15)^. This approach considers the diversities and complexities that govern different communicative contexts, including the historical processes of language formation and also the interpretations of signs and cultural symbols, present in the semiotics principles^(8)^.

In practice, this involves identifying and understanding the diverse languages ​​associated with ethnic identities that have entered Brazilian public universities, often as part of a process of reclaiming territory^(3,10,21)^. These ethnicities, linked to indigenous territories, are now part of the spaces of Higher Education Institutions (HEIs), bringing with them a diversity of worldviews^(3)^. At the semantic level, these expressions manifest themselves in different ways: in the deictic-referential model, they demand studies on the contexts and histories of these groups; and in the pragmatic sphere, they reflect indigenous bodies.

This pragmatics of indigenous bodies refers to the way they communicate and express meanings, through gestures, postures and specific cultural interactions, which vary according to sociocultural and historical contexts. Furthermore, there are semantic-phonetic perceptions, which encompass the interpretations of the meanings of words (semantics) and the sounds of languages (phonetics), by different peoples, which highlight relational aspects of their actions in the world^(8,9)^.

Therefore, the pragmatics of indigenous bodies involves specific cultural gestures, postures and interactions that are crucial to the nurse-patient relationship in multicultural contexts. However, these forms of expression are often ignored by training curricula based on colonial epistemologies, which reinforce the centrality of Brazilian Portuguese and thought patterns from the Global North. As a result, many non-indigenous professionals are unaware of cultural codes that could qualify and improve care, perpetuating communication barriers in relation to users, without excluding indigenous health practices^(3,17,22)^.

In this context, it is necessary to see the approximately 160 indigenous languages and dialects, spoken by the 305 ethnic groups present in Brazilian territory, which are organized into two language branches—*tupi* and *macro-jê*—, which comprise families and subgroups, with their peculiarities and diversities, according to the indigenous peoples who use them. *Tupi* is concentrated in most of the territories of Brazil, consisting of ten linguistic families, while *Macro-Jê* has nine families and is highly concentrated in the Legal Amazon area, but not only there. In addition, there are other isolated linguistic families, totaling 20 languages: *aikaná*; *arawá*; *arúak*; *guaikuru*; *iranxe*; *jabuti*; *kanoé*; *karib*; *katuxina*; *koazá*; *máku*; *makú/mura*; *nanambikwára*; *pano*; *trumái*; *tikúna*; *tukano*; *txapakúra*; and *yanomami* ^(22)^.

However, the lack of recognition of this linguistic plurality is a direct effect of the use of a colonial model that privileged Brazilian Portuguese and harmed native languages. This thinking needs to be revised, aiming to establish care in the native language of the people in question so that indigenous users are well received and can express themselves clearly, and healthcare professionals can successfully prescribe tests and/or medications.

Since communication takes place through language, when discussing indigenous peoples, it is necessary to understand them, based on their social histories and their linguistic roots, within the context of SASISUS and intercultural health^(2,15)^, observing how the semantic-phonetic perceptions of ethnolinguistic multiplicity^(18)^ impact healthcare processes. This conflicts with the fact that only Brazilian Portuguese is used to mediate processes in SUS, due to SASISUS’s reality, which incorporates different ethnicities in its administrative structure—the Special Indigenous Health Districts (In Portuguese, *Distritos Sanitários Especiais Indígenas* – DSEI)—, which are not evidenced in SUS work processes, which are generalist and have a biomedical bias^(11)^.

Hence, the predominance of Brazilian Portuguese as a mediator in SUS procedures disregards the linguistic diversity of indigenous peoples and creates significant barriers to healthcare for these groups, since the lack of translators or bilingual materials makes it difficult to transmit critical information in many scenarios within SASISUS. This nuance highlights a critical deficiency in training: the lack of appropriate technical and pedagogical approaches to care of indigenous peoples due to skill deficits, resulting in care that does not respect cultural and linguistic specificities. To overcome these limitations, it is essential to implement inclusive language policies that guarantee the production of bilingual materials, the training of cultural interpreters and the qualification of health teams to deal with the linguistic and cultural pluralities present in SUS contexts.

In this way, reflections are made on the different cultural contexts that can be identified, when conducting semiotic approaches to nursing, as verbal signs represent different ideas, according to the different language branches in use, currently^(22)^. Therefore, it is important to include, discuss and understand, in nurses’ training paths, the symbolic elements^(3)^ present in convergences and divergences that can be identified in indigenous peoples’ living spaces, by nursing teams, whether or not coinciding with the territories in which they operate, in particular those that have a direct relationship with rituals, which influence adherence to SUS medical care^(13)^.

In healthcare practice, this pragmatics is reflected in gestures and silences, which carry cultural meanings. For instance, silence may indicate consent rather than disinterest. Semantic-phonetic perceptions emerge when the translation of technical terms in the healthcare field, such as “virus,” acquires different meanings in indigenous languages, affecting the understanding of the diagnosis and the acceptance of treatment. This reveals the problems and scenarios faced by indigenous students when they enter higher education.

The Eurocentric hegemony of HEIs is particularly challenging and oppressive for these students, who face linguistic and epistemological challenges when faced with an education system that rarely recognizes or values their knowledge and ways of understanding the world. Acknowledging these conditions is essential for communication that transcends technical knowledge and is capable of contextualizing care. It is in this field that linguistics’ and semiotics’ contributions emerge as fundamental responses, as they offer theoretical and methodological tools that allow for the improvement of training and educational environments.

To implement the so-called intercultural education, it is essential that nursing courses incorporate specific modules in linguistics and semiotics, in order to develop communication skills that consider the cultural and linguistic pluralities of indigenous peoples. These disciplines can cover both theoretical aspects—introducing concepts of semantic analysis, pragmatics and semiotics—and simulated care practices, allowing students to practice anamnesis and health interviews in multicultural contexts, supporting a quality education^(19,20)^. Thus, upon completing their degree, graduate students will be able to promote inclusive actions when in contact with the particularities of healthcare for indigenous peoples.

On the other hand, studies on indigenous languages have been affected by structural issues^(9)^, discourses on intolerance and prejudices in learning processes^(10)^, aspects that point to the need to discuss this topic in the qualification of indigenous and non-indigenous nurses. When addressing this issue, some points can be mentioned to guide the debate: discourses on prejudice, intolerance and linguistic variety; production of non-inclusive instruments; teaching as a subject of social constructs; and the role of educational spaces in social inclusion^(3,12)^.

In this regard, we emphasized the implementation of cross- cutting proposals, addressing phenomena observed in different languages, translated into care, management and educational technologies, which have the potential to be used within SUS. These technologies mediate healthcare and education processes, promoting everything from training to the development of skills, through practices that aim at inclusive, identity-based and diverse products^(21)^, such as dictionaries, to mediate the care provided by nursing teams in DSEI.

The connection between linguistics and assistive technologies goes beyond the simple translation of words; these tools allow healthcare professionals to adapt their communications to indigenous communities’ cultural and linguistic specificities, helping to explain diagnoses and procedures, and to compose more accessible guidelines. This adaptation can provide descriptions of concepts in indigenous languages, avoiding misunderstandings, can promote more effective communication and can contribute to the strengthening of cultural identity and the symbolic and practical reintegration of indigenous territories into SASISUS.

Through this approach, it is possible to conduct structured training in inclusion and dialogues on linguistics and semiotics in pedagogical projects and instruments, both for the adequate transmission of information and for the construction of relationships of trust and empathy in care processes^(13,21)^. To this end, it is necessary to value individualities and promote relationships of mutual respect and understanding, updating and improving professionals’ communication skills^(13)^, from the moment students enter undergraduate courses.

Therefore, the inclusion of linguistics and semiotics in nursing training fosters the necessary mediations within SUS so that experiences in healthcare services are positive. Thus, the process must end by raising awareness among educational management, faculty and indigenous and non-indigenous students about this reality. This is a unique opportunity to implement studies on semiotics in product development^(21)^, for instance.

In nursing education, the discussions focused on a common goal: to promote equity and inclusion in healthcare in the work of SUS services. The education of professionals who are sensitive to indigenous peoples’ cultural and linguistic specificities is essential to overcoming the limitations of a system historically based on colonial epistemologies, observed throughout academic training^(23)^. At the same time, this change in the forms of action is reflected in movements of symbolic and practical reintegration of territories, reaffirming the centrality of indigenous worldviews in the construction of exercises and in the production of inclusive health technologies^(24)^. Thus, the articulation between the health and education dimensions^(24,25)^ represents a significant step towards transforming SUS into a truly intercultural system, aligned with indigenous peoples’ needs and social justice principles.

In terms of equity, the movement around the issue does not only achieve global targets^(1)^, especially those related to health and education^(25)^, but also constitutes a political instrument that affirms the restitution of territories^(3)^. On a global health scale, this represents equality of rights, differences and diversities, including providing opportunities to reduce the social inequalities listed in the different SDGs^(1)^.

### Translation and Interpretation of Languages: Paths Towards Equity in Healthcare

Language translation and interpretation play a fundamental role in promoting equity in SUS as well as overcoming language barriers in healthcare services^(4,5)^. On the other hand, when not overcome, such obstacles compromise diagnoses, adherence to treatments and health outcomes, reinforcing structural inequalities. In this sense, the inclusion of indigenous interpreters-translators is necessary to mediate communication and to respect the worldviews and cultural values of indigenous peoples, from the perspectives of interculturality, multilingualism^(18)^ and instrumentalization, through extension and research^(26)^.

Likewise, translation and interpretation are crucial to building trusting relationships, fostering a culture of inclusive communication that ensures patient safety^(5)^. On the other hand, the lack of translators-interpreters and effective public policies in this regard hinders access to fundamental rights, which grant human dignity to individuals. It is therefore essential to guarantee the presence of these literate professionals in the services provided to indigenous communities, in order to achieve the principle of equality, in which everyone can understand and be understood, a right endorsed by the Federal Constitution and Convention 169 of the International Labor Organization^(26,27,28)^.

Therefore, there are two points to highlight: the inclusion of linguistic dialogues in nursing training; and the hiring of interpreters-translators to mediate communications in SUS. The first broadens critical and reflective perspectives, since discussions and dialogues on diversity and indigenous worldviews have a sociopolitical role^(25)^, and the second highlights the role and place of languages^(25)^, since it brings them closer to the symbolic values of indigenous peoples, putting into practice the political character observed in the different ways of communicating^(8,9,25)^. In this way, the social function of inserting linguistic and semiotic paradigms into these contexts becomes clear, as they can build paths towards equity^(27)^.

Therefore, it is not enough to simply promote specific discussions on the value of linguistics and semiotics; it is necessary for these sciences to occupy a place and a function^(25)^. This occurs through curricular integrations, i.e., through the provision of training and certification programs that generate employment contracts in PHC, hospitals and clinics^(4,5)^, especially for those who are familiar with indigenous languages—in addition to nurses—, as it involves not only formative aspects, but also interactional, cultural, and other aspects^(25)^. This logic is in line with effectiveness and problem-solving, as well as relationships with others, which bring benefits to individuals and ratify nursing’s political commitment^(26,27)^.

The presence of interpreters is already foreseen, based on legal proceedings, upon request from the National Indigenous Foundation (In Portuguese, *Fundação Nacional do Indígenas* – FUNAI), which is in line with international and human rights. In the case of health, there are few records of this in actions by FUNAI and SASISUS, considering that the support of indigenous interpreters can be called upon to assist in the care provided to indigenous peoples, when it is necessary to leave their villages.

Furthermore, this change in mentality is essential to mediate the care provided by MIHT, made up of indigenous nurses who provide instructions on humanized care and health management^(26)^. However, some points should be considered, such as the integration of these workers into MIHT’s daily routine, with their shifts and shifts, in order to guarantee their availability. The participation of interpreters in all stages of care (from triage to discharge) deepens the understanding of the cultural and linguistic dimensions that affect diagnosis and treatment, since, in addition to translating technical terms, they act as a sociocultural mediator, promoting an intercultural practice.

In clinical contexts, the presence of interpreters and translators ensures effective communication that transcends simple linguistic translation, as it encompasses the interpretation of specific cultural contexts, values and practices^(25)^, as indicated in the global goals^(19,20)^. Inaccurate diagnoses and inadequate treatments can arise from misunderstandings resulting from the absence of interpretative bridges^(26)^, which not only translate words, but also cultural nuances and concepts of health and disease, which are intrinsic to indigenous worldviews^(29)^.

Furthermore, indigenous peoples have an intricate and deep relationship with their environments, and it is essential to properly interpret these connections. For instance, variations in river temperatures are not only ecological events for these communities, but can have direct implications for their health and well-being, as they can influence aquatic ecosystems’ health, impacting indigenous diets and medicines, and even their cultural practices. Here, the role of an interpreter goes beyond translation: they facilitate interdisciplinary understanding of the complex interactions between culture, health, and environment^(30)^.

While translators primarily deal with written materials, interpreters can work orally and directly during consultations and nursing procedures. In this sense, establishing work protocols that clearly define the responsibilities of each health communication professional is a starting point to avoid overlapping communications.

Mediation by interpreters who accompany indigenous people undergoing treatment in SUS favors communication for their adherence to treatments, as well as the effectiveness of the results, while, through the organization of indigenous families, there is a new generation of bilingual indigenous people who, in general, accompany these users. However, there is a repressed demand, which favors the promotion of these professionals beyond actions as nurses and indigenous nurses, since non- indigenous people can produce disparate and generic communications, due to the lack of knowledge of the cultures that characterize them.

This is stated because the linguistic organization of each ethnic group is unique and represents a particular cultural context, which didactically indicates that indigenous interpreters- translators’ work can result in a better understanding of the needs inherent to that ethnic group in relation to the dialogue carried out by non-indigenous personnel, who can present generic interpretations^(30)^. Within SUS, the incorporation of these interpreters can be essential in areas such as cleaning, security and disease, where terminology and practices specific to an ethnic group can be misinterpreted or oversimplified when translated by non-indigenous people. By implementing culturally competent translation, SUS can ensure more effective and adapted service delivery.

For SUS, this means access to information through the mother tongue, which recognizes and includes multilingualism. Considering the demands for equitable actions, effective translation makes communication possible. Therefore, it is important to recognize the different symbology that indigenous languages present in each community or not, since some signs cannot be translated. In any case, this thinking is capable of promoting better health, as well as implementing actions planned in health conferences, which recognize the needs for translation and interpretation in Healthcare Networks^(11)^.

In order to raise awareness of MIHT, there is a need for continuing education, i.e., healthcare professionals can receive ongoing training on how to work as interpreters and how to respect and accommodate patients’ linguistic needs. It is also possible to offer linguistic inclusion policies, which can be implemented to ensure that patients’ linguistic rights are respected^(25)^.

In this context, nurses’ and indigenous students’ fundamental importance as cultural and linguistic mediators capable of promoting effective communication stands out. As mentioned, these professionals face significant challenges during their academic training, especially those related to the health technical language, which represents an additional obstacle for those who do not have Brazilian Portuguese as their native language. However, learning Brazilian Portuguese and technical terminology in health can be implemented through tutoring programs, adapted teaching materials and bilingual glossaries.

It is appropriate to foresee partnerships between HEIs and indigenous leaders, in order to guarantee certification processes that recognize the linguistic and cultural skills inherent to work. Furthermore, ongoing training programs for nursing teams and other healthcare professionals are essential, as they reinforce the understanding of the importance of interculturality and multilingualism in clinical practice.

In this regard, the placement of interpreters and translators in SUS, combined with raising awareness among health teams about the cultural specificities of indigenous peoples, not only strengthens equity in care, but also advances towards SDG targets^(19,20)^. The presence of bilingual and culturally competent professionals also contributes directly to reducing inequalities (SDG 10), by facilitating indigenous peoples’ access to information and healthcare services as well as promoting quality health (SDG 3), by ensuring more accurate diagnoses and treatments that are more aligned with local worldviews. Thus, the adoption of policies and practices that recognize and include indigenous languages benefits the communities involved and reinforces global commitments to human dignity and social justice, based on inclusive education, as provided for in SDG 4^(19,20)^.

## FINAL CONSIDERATIONS

This study contributes to the ability to communicate inclusively in the field of indigenous health, based on linguistics and semiotics, seeking to understand the cultural nuances associated with this specific mode of communication, as such an exercise increases accessibility and inclusion of this public within SUS healthcare. Evidently, this research enriches knowledge production, through the rupture with the colonial epistemological bases that characterize the communication process described here.

The inclusion of specific linguistic and semiotic content in undergraduate courses, combined with the development of bilingual tools and the adoption of policies that respect the diverse indigenous worldviews, reinforces commitments to equity and social justice. In this scenario, the reintegration of territories and the recognition of indigenous peoples’ languages and cultural practices are not limited to specific actions, but become global health strategies that directly dialogue with the SDGs, especially those related to reducing inequalities, education and health promotion.

This reflection is limited to discussing the introduction of ideas from linguistics and semiotics among indigenous and non-indigenous nurses, and it is necessary to expand the discussion to the areas of other professionals who work in SUS, observing the hierarchies in health. Indigenous peoples face barriers in accessing culturally appropriate healthcare services within and outside their territories, but interculturality allows exchanges of knowledge between shamans, healers, blessers and midwives, whose healing traditions can be effective for all users of the territories.

Therefore, there is a need to conduct research on teaching qualifications, on monitoring indigenous and non-indigenous students’ learning, on the difficulties faced by indigenous nursing students in continuing their university courses, and on the development of care technologies with semiotic assessment, taking into account the different ethnicities under SUS care. Likewise, there is a demand for studies on indigenous interpreters-translators within SUS and on the construction and use of technologies, such as applications and dictionaries, to facilitate the interpretation and translation of dialogues with indigenous people assisted within the public health system, aiming at the creation of effective diagnoses that help professionals to provide more efficient care.

On the other hand, it is opportune to include research professors in spaces for dialogue on the topic, such as specific working groups in public bodies so that they can directly participate in the construction of policies that foster discussions in the area of ​​training. This can contribute to the development and insertion of inclusive instruments in training processes, in order to broaden the discussion in spaces of power in universities, such as graduate courses, which concentrate on the creation of Eurocentric knowledge.

## Data Availability

The database supporting this article is not publicy available.
