# Supplementary material for: Linguistics and semiotics in indigenous and non-indigenous people’s training and performance in nursing
Source: Rev Esc Enferm USP. 2025 Aug 4;59:e20250023. doi: 10.1590/1980-220X-REEUSP-2025-0023en (PMC12323388; doi:10.1590/1980-220X-REEUSP-2025-0023en)
Supplement: Translation

# Translation: A linguística e a semiótica na formação e atuação de indígenas e não indígenas na enfermagem

**DOI:** 10.1590/1980-220X-REEUSP-2025-0023pt

**Authors:** Nádile Juliane Costa de Castro, Purupramare Lima Gavião, Antônio Luis Parlandin dos Santos, Fernanda Teixeira Paes

**Affiliations:** 1 Universidade Federal do Pará, Programa de Pós-Graduação em Enfermagem, Belém, PA, Brasil.; 2 Ministério da Saúde, Secretaria de Saúde Indígena, Distrito Guamá. Tocantins, Belém, PA, Brasil.; 3 Universidade Federal do Pará, Faculdade de Letras. Belém, PA, Brasil.

**Keywords:** Aprendizagem, Enfermeiras e Enfermeiros, Povos Indígenas, Desenvolvimento de Pessoal, Diversidade, Equidade, Inclusão

## Abstract

**Objetivo:**

Refletir sobre as contribuições da linguística e da semiótica para a formação intercultural e para a atuação de enfermeiros e enfermeiras indígenas e não indígenas no Sistema Único de Saúde, destacando os desafios, as potencialidades e as estratégias de comunicação inclusiva nos cuidados em saúde de povos indígenas.

**Método:**

Estudo de abordagem teórico-reflexiva, sustentado na literatura contemporânea, assim como na experiência e na identidade de seus autores, a fim de elucidar os aportes trazidos por aquelas ciências, à luz da interculturalidade crítica.

**Resultados:**

Organizados em dois núcleos, seguindo a sequência de ideias sobre formação, sobre implementação e sobre ação: Linguística e semiótica na formação intercultural: estratégias para a equidade na atuação profissional; e Tradução e interpretação de línguas: caminhos para a equidade no cuidado em saúde.

**Considerações finais:**

A inserção de conteúdos específicos de linguística e de semiótica na graduação, associada ao desenvolvimento de ferramentas bilíngues e à adoção de políticas que respeitem as diversas cosmovisões indígenas, reforça os compromissos com a equidade e com a justiça social no atendimento em saúde do sistema público de saúde brasileiro.

## INTRODUÇÃO

A implementação de políticas de saúde e de educação passa por processos inclusivos^(1)^, como o do diálogo entre as diversidades linguística e cultural, nos espaços educacionais e nos serviços de saúde^(2)^. Esses processos se aproximam das discussões sobre a interculturalidade, contribuindo com apontamentos teóricos e práticos na formação e na atuação em saúde de indígenas enfermeiras e enfermeiros e de não indígenas. Tal fato se alinha, portanto, a ambientes de aprendizagem sustentáveis, inclusivos e equitativos^(1,3)^, alicerçados nos princípios do Sistema Único de Saúde (SUS)^(4,5)^ e nos Objetivos de Desenvolvimento Sustentável (ODS), principalmente os ODS 3 e 4 (Saúde e bem-estar e Educação de qualidade), respectivamente, conferindo agires crítico, reflexivo e cidadão às instituições educacionais.

Esse movimento contribui para a ampliação do corpo de conhecimentos e promove rupturas com as epistemologias eurocêntricas, paradigma central nas atuais formações, convergindo para a inclusão de epistemologias do Sul Global alinhadas às medicinas indígenas e às práticas de cuidado dos 305 povos indígenas presentes no território brasileiro^(3)^. Igualmente, essas mudanças fornecem subsídios teóricos às problematizações de peculiaridades étnicas e de necessidades em saúde dos territórios^(6)^, dentro das perspectivas de conceitos sobre saúde e sobre educação intercultural^(4,5)^. Além disso, colaboram para o enfrentamento de questões emergentes, em virtude dos seus movimentos multiepistêmicos, capazes de apreender novas nuanças pedagógicas e de empreender dialogicidades com as usualmente aplicadas.

No âmbito do SUS, esse cenário plural é identificado principalmente no Subsistema de Atenção à Saúde Indígena (SASISUS), que norteia os cuidados em saúde de povos indígenas^(7)^, o qual demanda uma abordagem política que verse sobre a inclusão e interdisciplinariedade^(2,3)^. Nesse sentido, as ciências das linguagens podem contribuir, por meio da linguística e da semiótica^(8)^, a fim de trazer concepções sobre as formas de comunicação das etnias atendidas pelo SASISUS, objetivando compreender símbolos agregados e identificar sentidos atribuídos a elementos nas culturas em questão^(9)^.

A enfermagem, enquanto protagonista deste cenário, demanda avanços na diversidade e na comunicação, importantes para o agir seguro e para as boas práticas nos cuidados em saúde^(4)^, que não resultem, consequentemente, em diagnósticos imprecisos, em tratamentos inadequados e em desfechos de saúde desfavoráveis^(7)^. Portanto, este estudo se justifica pelas suas proposições de preencher lacunas de conhecimentos, referentes à subutilização das ciências da linguagem nas comunicações com os povos indígenas, e de compor uma resposta à Política Nacional de Atenção à Saúde dos Povos Indígenas.

Além disso, o presente estado é fundamental em seu alinhamento epistemológico com autores indígenas, que reconhecem as pluralidades étnica, cultural e das cosmovisões de suas sociedades. Esta discussão demonstra, ainda, a indissociabilidade entre as formações para atuação em territórios indígenas e as demais áreas do conhecimento, como a da linguística, que mediam a comunicação entre usuários e Equipes Multidisciplinares de Saúde Indígena (EMSI), desde a Atenção Primária à Saúde (APS)^(7)^, em que se encontram enfermeiros e enfermeiras, médicos, agentes indígenas de saúde, entre outros.

Estudos evidenciam, nessa direção, os desafios à comunicação entre as EMSI e os pacientes indígenas; desafios que ocorrem dentro e fora dos territórios, na APS e em outros níveis de atenção à saúde, associados sobretudo às formações na graduação. Por outro lado, tais oportunidades podem trazer excelentes contribuições, em virtude do acesso de indígenas ao ensino superior, a partir da Lei n.º 14.723/2023 (Lei de Cotas), que possibilita a formação de indígenas enfermeiros e enfermeiras.

No entanto, considerando as relações entre estudantes indígenas e não indígenas, esse acesso deve ter acompanhamentos pedagógicos, interpretações linguísticas e semióticas, e comunicações inclusivas, apreensões sistêmicas ainda poucos exploradas^(7,10)^. Tal aspecto salienta outras questões da formação, como a superação da intolerância e o preconceito linguístico, o que demanda mudanças epistemológicas, no sentido de possibilitar intervenções na prática social em saúde, visando alcançar a atenção diferenciada^(11-13)^.

Essas intervenções promovem a equidade na mediação dos serviços de saúde, moldam novas estruturas e infraestruturas^(4)^ no âmbito dos serviços de saúde, e impulsionam pesquisas que enriquecem a compreensão das necessidades das formações para atuação no SASISUS^(4,11)^, contribuindo para diminuir as iniquidades sociais e de saúde, em vista da promoção de qualificações inclusivas e socialmente engajadas^(1)^.

Nesse sentido, questionaram-se quais as melhores estratégias a serem utilizadas para qualificar os processos de aprendizagem na formação de enfermeiros e enfermeiras indígenas e não indígenas, bem como na atuação desses com usuários indígenas, com a finalidade de apontar limitações e possibilidades de implementação de processos de aprendizagem e de cuidado. Somam-se a esses os construtos que podem ser desenvolvidos transversalmente, pela extensão e pela pesquisa, e as estratégias de intervenção que podem/devem ser empreendidas, para promover práticas efetivas de cuidados em saúde para os povos indígenas. Enfim, este estudo tem como objetivo refletir sobre as contribuições da linguística e da semiótica para a formação intercultural e atuação de enfermeiros e enfermeiras indígenas no SUS, destacando os desafios, as potencialidades e as estratégias de comunicação inclusiva nos cuidados em saúde de povos indígenas.

A linguística e a semiótica fornecem ferramentas essenciais para compreender os processos comunicativos, especialmente em contextos interculturais e multilíngues, como é o caso da formação de enfermeiros e enfermeiras que atuam com povos indígenas. Esses campos possibilitam abordagens crítica e reflexiva sobre os modos pelos quais a linguagem e os símbolos moldam as relações de cuidado em saúde, contribuindo diretamente para a formação de profissionais culturalmente competentes. Neste estudo, as reflexões serão conduzidas, a partir de diálogo com autores do Sul Global, observando estudos sobre saúde intercultural, que têm norteado as discussões sobre atenção à saúde indígena, no sentido de possibilitar a dialogicidade entre as identidades dos autores indígenas e as formações acadêmicas.

### Aspectos Conceituais da Linguística e da Semiótica e Perspectivas Indígenas

A linguística é a ciência que estuda a linguagem verbal em suas múltiplas dimensões, contemplando aspectos descritivos, históricos e teóricos da língua, como sistema estruturado de comunicação, e suas constantes elaboração e reelaboração^(8)^. Essa se diferencia da semiótica, que se dedica ao estudo de signos, abrangendo sistemas e processos representados por símbolos e por indícios^(14)^, em suas formas, tipos e efeitos. Enquanto a linguística focaliza as estruturas e o funcionamento das línguas, em suas dimensões dêitico-referencial, lógica, pragmático- discursiva, sintática e semântica^(15)^, a semiótica investiga como os significados são construídos, compartilhados e interpretados, por meio dos diferentes sistemas de signos presentes nas diversas culturas^(14)^.

Nesse sentido, reconhecer as subáreas da linguística, como semântica, pragmática e sociolinguística, possibilita compreender significados, usos da língua em determinados contextos e relações sociais, respectivamente^(8)^. Além disso, distinguir a função da semiótica cultural é fundamental ao estudo dos signos em contextos indígenas, em virtude da multimodalidade (verbal, visual, gestual, espacial) neles identificada e da incorporação de diferentes elementos à análise das práticas simbólicas das diferentes etnias^(16,17)^. Essa separação é fundamental para compreender como os campos do conhecimento contribuem diferentemente para a formação intercultural em saúde, especialmente em contextos multilíngues^(9)^.

Por outro lado, é importante ressaltar que a linguística e a semiótica têm suas bases conceituais e metodológicas construídas em epistemológicos ocidentais^(8,14)^. Nesse sentido, há riscos ao aplicar os conceitos eurocêntricos que as caracterizam às realidades indígenas sem a devida problematização, pois os povos indígenas do território brasileiro possuem seus próprios sistemas de produção e de interpretação de significados, intrinsecamente conectados às suas cosmovisões^(16)^. Portanto, é necessário observar uma “semiótica indígena” que reconheça sistemas próprios de signos e de significações, que se manifestam através de pinturas corporais, de grafismos, de cantos, de narrativas, de rituais e de relações próprias com o território^(17)^.

## MÉTODO

Trata-se de estudo teórico-reflexivo, sustentado na literatura científica sobre linguística e sobre semiótica, referente à formação de enfermeiros e enfermeiras indígenas e não indígenas, assim como nas percepções, nas experiências e nas identidades de seus autores, a fim de elucidar as relações estabelecidas entre a instrução e a atuação desses profissionais. Esta pesquisa foi realizada entre novembro de 2023 e dezembro de 2024, e teve sua base na chamada educação intercultural, de Gersen Baniwa^(16)^, através da qual o autor afirma que é necessário um olhar crítico sobre as epistemologias que têm sido aplicadas nos processos formativos, sendo imprescindíveis a ruptura dessa hegemonia e a condução de diálogos com outros modos de pensar.

As qualificações linguística e cultural são centrais e subsidiam melhorias na eficácia comunicacional da interação entre enfermeiros e enfermeiras e pacientes indígenas^(18)^, aspecto que, à luz do pensamento e da linguagem, transcende a compreensão dos fenômenos sociais. Na formação desses trabalhadores, tais melhorias apresentam potencial para o desenvolvimento de ações interculturais complexas^(2)^, tanto no nível social—em um primeiro momento—quanto no individual—em momento posterior^(14)^—, decisivas para entender e para aprimorar a comunicação e o aprendizado em contextos de saúde multiculturais e multilíngues^(8,9)^ de atuação.

Para que a formação de enfermeiros e enfermeiras seja inclusiva, defende-se que seja mediada por conceitos de caráter indigenista da linguística e da semiótica^(17)^, o que deve contribuir para a promoção de práticas comunicativas mais abrangentes, para a redução de desafios linguísticos e culturais no âmbito do SUS, e para o fortalecimento de políticas públicas orientadas à equidade. Tal paradigma também pode subsidiar aprendizagens mais holísticas na enfermagem^(19)^ e metas presentes nos ODS 3 e 4, notadamente o desenvolvimento e a formação de profissionais na área da saúde (item 3c), e a valorização da diversidade cultural (item 4.7)^(19,20)^.

Este texto está estruturado em dois núcleos temáticos: Linguística e semiótica na formação intercultural: estratégias para a equidade na atuação profissional; e Tradução e interpretação de línguas: caminhos para a equidade no cuidado em saúde. Trata-se de trabalho de cunho teórico-reflexivo, não havendo necessidade de avaliação pelo sistema CEP/CONEP.

### Desenvolvimento

Como ponto de partida, é necessário compreender a linguagem como instrumento técnico e como meio de construir relações interculturais, mediadas por símbolos, por valores e por contextos específicos de comunidades indígenas. Nesse sentido, a partir das experiências dos autores, é possível identificar desafios e potencialidades, para apontar estratégias pragmáticas.

A partir da dialogicidade proposta, entende-se que a interação social é central no desenvolvimento do pensamento e da linguagem^(16)^, refletindo diretamente na formação de profissionais de saúde. Por outro lado, a questão envolve outros aspectos, tais como a identificação das diversidade étnica^(16)^, dos territórios indígenas e dos equipamentos em saúde e suas tipologias (Figura 1), a fim de que os profissionais indígenas e não indígenas recebam uma educação que valorize as línguas maternas e que compreenda os significados simbólicos atribuídos à saúde e à doença por cada grupo — e as implicações desses nos processos de cuidado.

**Figura 1:** Representação da dialogicidade na formação intercultural de enfermeiros e enfermeiras.

Fonte: autores, 2025.

### Linguística e Semiótica na Formação Intercultural: Estratégias para a Equidade na Atuação Profissional

A competência intercultural^(10,15)^ e o reconhecimento do multilinguismo são decisivos para enfermeiros e enfermeiras, especialmente para aqueles que atuam no SASISUS, em regiões com diferentes povos indígenas. Tais aspectos são fundamentais à aquisição de cuidados em saúde equitativos^(4,5)^, cujas habilidades de identificar e de navegar por várias línguas ampliam a compreensão das nuanças culturais^(4)^, que são intrínsecas à comunicação, e a interpretação das mensagens em saúde^(13)^. Além disso, o estabelecimento de uma comunicação com sensibilidade cultural enriquece a interação paciente-enfermeiro^(5)^ e também promove uma abordagem mais holística, em termos de cuidado^(13)^.

O aprimoramento na comunicação engloba as aptidões para compreender e para ser compreendido nos espectros multilíngue^(18)^ e multicultural, intrínsecos à eficácia e à qualidade do atendimento prestado no âmbito do SUS, o que transcende a simples transmissão de informações. Igualmente, esse melhoramento emerge como pilar fundamental à valorização da diversidade cultural, reconhecendo que cada indivíduo traz consigo saberes e práticas que moldam sua percepção de saúde, como apontado nos ODS^(19,20)^. Quando bem estruturada, tal melhoria pode instrumentalizar enfermeiros e enfermeiras quanto à adequada transferência de conhecimentos críticos à saúde^(5)^, à construção de relações de confiança, de empatia, e à correta sistematização dos cuidados necessários ao chamado atendimento humanizado, principalmente no SASISUS^(10,13)^.

Além disso, as capacidades de se comunicar e de compreender nuanças culturais ampliam a acessibilidade e a inclusão no âmbito dos cuidados em saúde^(2,13)^, facilitando a implementação de estratégias de promoção da saúde e de prevenção de doenças que respeitem as singularidades de cada etnia^(13)^. Dessa maneira, na interface entre linguística, semiótica e equidade^(8,9)^, aponta-se que a inclusão de pessoas indígenas em diferentes níveis de educação, como graduação e pós-graduação^(1,3)^, é ponte para o desenvolvimento profissional^(19,20)^ e para o fortalecimento das representatividades cultural e epistêmica nos espaços educacionais.

Outrossim, tal aperfeiçoamento pode promover a construção de práticas mais humanizadas e mais respeitosas de cuidados, alinhadas às especificidades e às cosmovisões indígenas, além de contribuir para a redução das iniquidades em saúde e para a consolidação de uma formação crítica e socialmente justa no SUS. Para tanto, é necessário inserir esses paradigmas nos processos educacionais e reconhecer o papel fundamental da linguística na condução do processo, pois a mesma é baseada nos recursos que analisam suas relações estruturais^(5)^, abrangendo as dimensões semântica, dêitico-referencial e pragmático-discursiva^(8,9,15)^. Essa abordagem considera as diversidades e as complexidades que regem os diferentes contextos comunicativos, incluindo os processos históricos de formação das línguas e, também, as interpretações de signos e de símbolos culturais, presentes nos princípios da semiótica^(8)^.

Na prática, isso implica identificar e compreender as diversas linguagens, associadas às identidades étnicas, que têm ingressado nas universidades públicas brasileiras, muitas vezes como parte de processo de retomada de território^(3,10,21)^. Essas etnias, vinculadas a territórios indígenas, integram agora os espaços das Instituições de Ensino Superior (IES), trazendo consigo uma diversidade de cosmovisões^(3)^. No plano semântico, essas expressões se manifestam de formas variadas: no modelo dêitico-referencial, demandam estudos sobre os contextos e sobre as histórias desses grupos; e no âmbito pragmático, refletem os corpos indígenas.

Tal pragmática dos corpos indígenas se refere à forma como eles comunicam e expressam significados, por meio de gestos, de posturas e de interações culturais específicas, que variam conforme os contextos socioculturais e históricos. Além disso, há as percepções semântico-fonéticas, que englobam as interpretações dos significados das palavras (semântica) e dos sons das línguas (fonética), pelos diferentes povos, as quais evidenciam aspectos relacionais de seus agires no mundo^(8,9)^.

Portanto, a pragmática dos corpos indígenas envolve gestos, posturas e interações culturais específicas cruciais à relação enfermeiro-paciente em contextos multiculturais. No entanto, essas formas de expressão costumam ser ignoradas por currículos de formação pautados em epistemologias coloniais, que reforçam as centralidades do português e dos padrões de pensamento do Norte Global. Como resultado, muitos profissionais não indígenas desconhecem códigos culturais que poderiam qualificar e melhorar o cuidado, perpetuando barreiras de comunicação em relação aos usuários, sem a exclusão de práticas indígenas de saúde^(3,17,22)^.

Nessa conjuntura, é necessário enxergar as cerca de 160 línguas indígenas e dialetos, falados pelas 305 etnias presentes no território brasileiro, as quais são organizadas em dois troncos linguísticos—o tupi e o macro-jê—, que compreendem famílias e subgrupos, com suas peculiaridades e diversidades, conforme os povos indígenas que as empregam. O tupi se concentra na maior parte dos territórios do Brasil, sendo constituído por dez famílias linguísticas, enquanto o macro-jê possui nove famílias e apresenta grande concentração na área da Amazônia Legal, mas não somente nessa. Além disso, há outras famílias linguísticas isoladas, totalizando 20 línguas: aikaná; arawá; arúak; guaikuru; iranxe; jabuti; kanoé; karib; katuxina; koazá; máku; makú/mura; nanambikwára; pano; trumái; tikúna; tukano; txapakúra; e yanomami^(22)^.

Todavia, a falta de reconhecimento desta pluralidade linguística é efeito direto do emprego de um modelo colonial que privilegiou a língua portuguesa e que prejudicou as línguas originárias. Tal pensamento precisa ser revisto, objetivando o estabelecimento de um atendimento na língua materna do povo em atenção, para que usuários indígenas tenham um bom acolhimento e consigam se expressar com clareza, de modo que o profissional de saúde tenha êxito nas prescrições de exames e/ou de medicações.

Como é por meio da língua que a comunicação se realiza, ao dialogar sobre povos indígenas, é necessário compreendê-los, a partir de suas histórias sociais e de seus troncos linguísticos, dentro das conjunturas do SASISUS e da saúde intercultural^(2,15)^, observando como as percepções semântico-fonéticas da multiplicidade etnolinguística^(18)^ impactam os processos de cuidado em saúde. Isso conflita com o fato de se usar somente a língua portuguesa para mediar os processos no SUS, devido à realidade do SASISUS, que incorpora diferentes etnias em sua estrutura administrativa—os Distritos Sanitários Especiais Indígenas (DSEI)—, as quais não são evidenciadas nos processos de trabalho do SUS, generalistas e de viés biomédico^(11)^.

Nesse sentido, a predominância da língua portuguesa como mediadora nos procedimentos do SUS desconsidera a diversidade linguística dos povos indígenas e gera barreiras significativas aos cuidados em saúde para esses grupos, pois a ausência de tradutores ou materiais bilíngues dificulta a transmissão de informações críticas em muitos cenários no âmbito do SASISUS. Tal nuança evidencia uma deficiência crítica na formação: a ausência de abordagens técnica e pedagógica adequadas ao cuidado de povos indígenas, em virtude de défices de habilidades, resultando em atendimentos que não respeitam especificidades culturais e linguísticas. Para superar essas limitações, é imprescindível a implementação de políticas linguísticas inclusivas que garantam a produção de materiais bilíngues, a formação de intérpretes culturais e a capacitação das equipes de saúde para lidar com as pluralidades linguística e cultural presentes nos contextos do SUS.

Nesse caminho, fazem-se reflexões sobre os diferentes contextos culturais que podem ser identificados, ao conduzir abordagens semióticas da enfermagem, conforme os signos verbais representam diferentes ideias, de acordo com os diversos troncos linguísticos em uso, atualmente^(22)^. Portanto, interessa incluir, discutir e compreender, nos percursos de instrução de enfermeiros e enfermeiras, os elementos simbólicos^(3)^ presentes nas convergências e nas divergências que podem ser identificadas nos espaços de vivência dos povos indígenas, pelas equipes de enfermagem, coincidentes ou não com os territórios de atuação dessas, em particular aqueles que possuem relação direta com rituais, que influenciam a adesão aos cuidados médicos do SUS^(13)^.

Na prática da saúde, essa pragmática se reflete em gestos e em silêncios, que carregam significados culturais. Por exemplo, o silêncio pode indicar consentimento em vez de desinteresse. Já as percepções semântico-fonéticas emergem quando a tradução de termos técnicos da área da saúde, como o de “vírus”, adquire significados distintos nas línguas indígenas, afetando a compreensão do diagnóstico e a aceitação do tratamento. Isso revela as problemáticas e os cenários enfrentados pelos estudantes indígenas, ao ingressarem no ensino superior.

A hegemonia eurocentrada das IES se mostra particularmente desafiadora e opressora para esses estudantes, que enfrentam desafios linguísticos e epistemológicos, ao se depararem com um sistema de ensino que raramente reconhece ou valoriza seus conhecimentos e suas formas de compreender o mundo. Reconhecer essas condições é essencial a uma comunicação que transcenda o conhecimento técnico e que seja capaz de contextualizar os cuidados. É neste terreno que as contribuições da linguística e da semiótica emergem como respostas fundamentais, pois oferecem ferramentas teóricas e metodológicas que permitem aprimorar as formações e os ambientes educacionais.

Para efetivar a chamada formação intercultural, é essencial que as graduações em enfermagem incorporem módulos específicos de linguística e de semiótica, de modo a desenvolver competências comunicativas que considerem as pluralidades cultural e linguística dos povos indígenas. Essas disciplinas podem contemplar tanto aspectos teóricos—introduzindo conceitos de análise semântica, de pragmática e de semiótica—quanto práticas simuladas de atendimento, permitindo que os estudantes exercitem a anamnese e a entrevista de saúde em contextos multiculturais, dando sustentação a uma educação de qualidade^(19,20)^. Desse modo, ao concluir a graduação, os egressos estarão aptos a promover ações inclusivas, quando do contato com as particularidades da atenção em saúde dos povos indígenas.

Por outro lado, os estudos sobre línguas indígenas têm sido atravessados por questões estruturais^(9)^, por discursos sobre intolerância e por preconceitos nos processos de aprendizagem^(10)^, aspectos que apontam a necessidade de dialogar sobre este tema na qualificação de enfermeiros e enfermeiras indígenas e não indígenas. Ao tratar da questão, podem-se mencionar alguns pontos para o direcionamento do debate: discursos sobre preconceito, sobre intolerância e sobre variedade linguística; produção de instrumentos não inclusivos; docência como sujeito de construtos sociais; e o papel dos espaços educativos na inserção social^(3,12)^.

Nesse caminho, frisa-se a implementação de propostas transversais, com abordagem dos fenômenos observados nas diferentes línguas, traduzidos em tecnologias assistenciais, gerenciais e educacionais, as quais têm potencial de utilização no âmbito do SUS. Essas tecnologias mediam processos de cuidado e de educação em saúde, promovendo desde a formação ao desenvolvimento de competências, por meio de práticas que objetivem produtos inclusivos, identitários e diversos^(21)^, como dicionários, para mediar as assistências das equipes de enfermagem nos DSEI.

A conexão entre linguística e tecnologias assistenciais vai além da simples tradução de palavras; essas ferramentas permitem que profissionais de saúde adaptem suas comunicações às especificidades culturais e linguísticas das comunidades indígenas, auxiliando na explicação de diagnósticos e de procedimentos, e na composição de orientações mais acessíveis. Essa adaptação pode trazer descrições de conceitos nas línguas indígenas, evitando mal-entendidos, pode promover uma comunicação mais eficaz e pode contribuir para o fortalecimento da identidade cultural e para as reintegrações simbólica e prática de territórios indígenas ao âmbito do SASISUS.

Por esta abordagem, é possível conduzir uma formação estruturada na inclusão e nos diálogos sobre linguística e sobre semiótica em projetos pedagógicos e em instrumentos, tanto para a transmissão adequada de informações quanto para a construção de relações de confiança e de empatia nos processos de cuidado^(13,21)^. Para tanto, é necessário valorizar as individualidades e promover relações de respeito e de entendimento mútuos, atualizando e aprimorando as competências comunicativas dos profissionais^(13)^, desde a entrada dos estudantes na graduação.

Portanto, a inclusão da linguística e da semiótica nas formações em enfermagem fomenta as mediações necessárias no âmbito do SUS, para que as experiências nos serviços de saúde sejam positivas. Assim, o processo deve encerrar as sensibilizações da gestão educacional, do corpo docente e dos estudantes indígenas e não indígenas sobre esta realidade. Essa é uma oportunidade ímpar para implementar estudos sobre semiótica no desenvolvimento de produtos^(21)^, por exemplo.

No ensino de enfermeiros e enfermeiras, as discussões enfocadas convergem para um objetivo comum: promover a equidade e a inclusão no cuidado em saúde na atuação nos serviços do SUS. A escolarização de profissionais sensíveis às especificidades culturais e linguísticas dos povos indígenas é essencial à superação das limitações de um sistema historicamente embasado em epistemologias coloniais, observadas em toda a formação acadêmica^(23)^. Simultaneamente, essa alteração nas formas de ações se reflete em movimentos de reintegração simbólica e prática de territórios, reafirmando a centralidade das cosmovisões indígenas na construção de exercícios e na produção de tecnologias inclusivas em saúde^(24)^. Assim, a articulação entre as dimensões saúde e educação^(24,25)^ configura um passo significativo para transformar o SUS em um sistema verdadeiramente intercultural, alinhado às necessidades dos povos indígenas e aos princípios da justiça social.

Na interface com a equidade, o movimento em torno do tema não alcança apenas metas globais^(1)^, principalmente as relacionadas à saúde e à educação^(25)^, mas constitui um instrumento político, o qual afirma a restituição de territórios^(3)^. Em uma escala de saúde global, tal representa igualdade de direitos, de diferenças e de diversidades, inclusive dando oportunidades para reduzir as desigualdades sociais elencadas nos diferentes ODS^(1)^.

### Tradução e Interpretação de Línguas: Caminhos Para a Equidade no Cuidadoem Saúde

A tradução e a interpretação de línguas desempenham um papel fundamental na promoção da equidade no SUS, bem como podem superar barreiras linguísticas nos serviços de saúde^(4,5)^. Por outro lado, quando não superadas, tais obstáculos comprometem diagnósticos, adesões a tratamentos e resultados em saúde, reforçando desigualdades estruturais. Nesse sentido, a inclusão de intérpretes-tradutores indígenas é necessária para mediar a comunicação e para respeitar as cosmovisões e os valores culturais dos povos indígenas, a partir das perspectivas da interculturalidade, do multilinguismo^(18)^ e da instrumentalização, por meio da extensão e da pesquisa^(26)^.

Da mesma forma, tradução e interpretação são cruciais à construção de relações de confiança, conferindo cultura de comunicação inclusiva que garante segurança ao paciente^(5)^. Por outro lado, a ausência de tradutores-intérpretes e de políticas públicas efetivas neste sentido dificulta o acesso a direitos fundamentais, que conferem dignidade humana aos indivíduos. É indispensável, portanto, garantir a presença desses profissionais letrados nos atendimentos às comunidades indígenas, a fim de alcançar o princípio da igualdade, em que todos possam compreender e ser compreendidos, direito referendado pela Constituição Federal e pela Convenção n.º 169 da Organização Internacional do Trabalho^(26-28)^.

Por conseguinte, há dois pontos a destacar: a inclusão de diálogos linguísticos na formação de enfermeiros e enfermeiras; e a contratação de intérpretes-tradutores para mediar as comunicações no SUS. O primeiro amplia os olhares crítico e reflexivo, uma vez que as discussões e os diálogos sobre diversidades e sobre cosmovisões indígenas têm um papel sociopolítico^(25)^, e o segundo evidencia o papel e o lugar das línguas^(25)^, uma vez que as aproxima dos valores simbólicos dos povos indígenas, colocando em prática o caráter político observado nas diferentes formas de se comunicar^(8,9,25)^. Desse modo, fica clara a função social das inserções de paradigmas da linguística e da semiótica nestes contextos, pois podem construir caminhos para a equidade^(27)^.

Por isto, não basta apenas promover discussões pontuais de valorização da linguística e da semiótica; é necessário que essas ciências ocupem um lugar e uma função^(25)^. Isso ocorre pelas integrações curriculares dessas, isto é, pela oferta de programas de treinamento e de certificação que gerem contratos de trabalho em APS, em hospitais e em clínicas^(4,5)^, especialmente para aqueles que estão familiarizados com as línguas indígenas—além dos enfermeiros e das enfermeiras—, pois não envolve somente aspectos formativos, mas os interacionais, os culturais, entre outros^(25)^. Tal lógica vai ao encontro da efetividade e da resolutividade, assim como da relação com o outro, que trazem benefícios aos indivíduos e que ratificam o compromisso político da enfermagem^(26,27)^.

A presença de intérpretes já é prevista, a partir de processos judiciais, mediante solicitação da Fundação Nacional do Indígenas (FUNAI), o que vai ao encontro de direitos internacionais e humanos. No caso da saúde, há poucos registros sobre isso em ações da FUNAI e do SASISUS, considerando que o suporte de intérpretes indígenas pode ser acionado para auxiliar nos cuidados prestados a povos indígenas, quando da necessidade de saída das aldeias.

Outrossim, essa mudança de mentalidade é fundamental para mediar o atendimento das EMSI, compostas por enfermeiros e enfermeiras indígenas que conferem instruções de cuidados e de gestão de saúde humanizados^(26)^. No entanto, alguns pontos devem ser pensados, como o da integração desses trabalhadores ao cotidiano das EMSI, com suas escalas e plantões, a fim de garantir sua disponibilidade. A participação do intérprete em todas as etapas do atendimento (da triagem à alta) aprofunda a compreensão das dimensões culturais e linguísticas que afetam o diagnóstico e o tratamento, uma vez que, além de traduzir termos técnicos, ele atua como mediador sociocultural, promovendo uma prática intercultural.

Em contextos clínicos, a presença de intérpretes e tradutores garante uma comunicação eficaz que transcende a simples tradução linguística, pois engloba a interpretação de contextos, de valores e de práticas culturais específicas^(25)^, como apontado nas metas globais^(19,20)^. Diagnósticos imprecisos e tratamentos inadequados podem surgir de mal-entendidos decorrentes da ausência de pontes interpretativas^(26)^, as quais não só traduzem palavras, mas também nuanças culturais e conceitos de saúde e de doença, que são intrínsecos às cosmovisões indígenas^(29)^.

Além disso, os povos indígenas possuem uma relação intrincada e profunda com seus ambientes, sendo essencial à interpretação adequada destas conexões. Por exemplo, variações na temperatura dos rios não são apenas eventos ecológicos para estas comunidades, mas podem ter implicações diretas em sua saúde e em seu bem-estar, pois pode influenciar a saúde dos ecossistemas aquáticos, repercutindo na dieta e na medicina indígenas e, mesmo, nas práticas culturais desses povos. Aqui, o papel de um intérprete vai além da tradução: ele facilita a compreensão interdisciplinar das interações complexas entre cultura, saúde e ambiente^(30)^.

Enquanto os tradutores lidam prioritariamente com materiais escritos, os intérpretes podem atuar de formas oral e imediata durante consultas e procedimentos de enfermagem. Nesse sentido, o estabelecimento de protocolos de trabalho, que definam claramente as responsabilidades de cada profissional no âmbito da comunicação em saúde, é um ponto inicial para evitar sobreposições de comunicações.

A mediação por intérpretes que acompanham indígenas em tratamento no SUS favorece a comunicação para a adesão desses aos tratamentos, assim como a eficácia dos resultados, ao passo que, por organização das famílias indígenas, verifica-se uma nova geração de indígenas bilíngues que, em geral, acompanham esses usuários. No entanto, existe uma demanda reprimida, que favorece a promoção desses profissionais para além de ações como enfermeiros e como enfermeiras indígenas, uma vez que não indígenas podem produzir comunicações dispares e genéricas, pelo déficit de conhecimento das culturas que os caracteriza.

Afirma-se isto, porque a organização linguística de cada etnia é singular e representa um contexto cultural particular, o que didaticamente aponta que a atuação de intérpretes-tradutores indígenas pode resultar em uma melhor compreensão das necessidades inerentes àquela etnia relativamente à interlocução feita por pessoal não indígena, que pode apresentar interpretações genéricas^(30)^. No âmbito do SUS, a incorporação desses intérpretes pode ser essencial a áreas, como as da limpeza, da segurança e das doenças, em que terminologias e práticas específicas de uma etnia podem ser mal interpretadas ou simplificadas demais, quando traduzidas por não indígenas. Ao implementar a tradução culturalmente competente, o SUS pode garantir uma prestação de serviços mais eficaz e mais adaptada.

Para o SUS, isso representa acesso a informações pela via da língua materna, que reconhece e que inclui o multilinguismo. Em vista das demandas por ações equitativas, a tradução efetiva torna a comunicação possível. Observa-se, portanto, a importância de reconhecer as diferentes simbologias que as línguas indígenas apresentam em cada comunidade ou não, pois alguns signos não podem ser traduzidos. De todo modo, tal pensamento é capaz de promover mais saúde, assim como implementar ações previstas nas conferências de saúde, que reconhecem as necessidades de tradução e de interpretação nas Redes de Atenção à Saúde^(11)^.

No sentido de sensibilizar as EMSI, verifica-se a necessidade de educação continuada, ou seja, os profissionais de saúde podem receber treinamento contínuo sobre o trabalho como intérpretes e sobre como respeitar e acomodar as necessidades linguísticas dos pacientes. É possível, também, ofertar políticas de inclusão linguística, que podem ser implementadas para garantir que os direitos linguísticos dos pacientes sejam respeitados^(25)^.

Nesse contexto, destaca-se a importância fundamental dos enfermeiros e enfermeiras e dos estudantes indígenas como mediadores culturais e linguísticos capazes de promover uma comunicação efetiva. Como mencionado, esses profissionais enfrentam desafios significativos durante suas formações acadêmicas, especialmente os relacionados à linguagem técnica da saúde, que representa um obstáculo adicional àqueles que não têm o português como língua materna. No entanto, o aprendizado da língua portuguesa e da terminologia técnica em saúde pode ser implementado com programas de tutoria, com materiais didáticos adaptados e com glossários bilíngues.

É oportuno prever parcerias entre IES e lideranças indígenas, a fim de garantir processos de certificação que reconheçam as competências linguísticas e culturais inerentes ao trabalho. Além disso, programas de capacitação permanente para as equipes de enfermagem e para os demais profissionais de saúde são fundamentais, pois reforçam a compreensão sobre a importância da interculturalidade e do multilinguismo na prática clínica.

Nesse sentido, a colocação de intérpretes e de tradutores no SUS, aliada à sensibilização das equipes de saúde para as especificidades culturais dos povos indígenas, não apenas fortalece a equidade no cuidado, mas também avança em direção às metas das ODS^(19,20)^. A presença de profissionais bilíngues e culturalmente competentes também contribui diretamente para a redução das desigualdades (ODS 10), ao facilitar o acesso dos povos indígenas à informação e aos serviços de saúde, bem como para a promoção de saúde de qualidade (ODS 3), ao garantir diagnósticos mais precisos e tratamentos mais alinhados às cosmovisões locais. Assim, a adoção de políticas e de práticas que reconheçam e que incluam as línguas indígenas beneficia as comunidades envolvidas e reforça os compromissos globais com a dignidade humana e com a justiça social, a partir da educação inclusiva, conforme previsto na ODS 4^(19,20)^.

## Considerações Finais

Este estudo traz contribuições sobre a capacidade de se comunicar de forma inclusiva no terreno da saúde indígena, com base na linguística e na semiótica, buscando compreender as nuanças culturais associadas a este modo específico de comunicação, na medida em que tal exercício amplia a acessibilidade e a inclusão deste público no âmbito dos cuidados em saúde do SUS. Evidentemente, esta investigação enriquece a produção do conhecimento, por meio da ruptura com as bases epistemológicas coloniais, que caracterizam o processo comunicacional aqui descrito.

A inserção de conteúdos específicos de linguística e de semiótica na graduação, associada ao desenvolvimento de ferramentas bilíngues e à adoção de políticas que respeitem as diversas cosmovisões indígenas, reforça os compromissos com a equidade e com a justiça social. Nesse cenário, a reintegração de territórios e o reconhecimento das línguas e das práticas culturais dos povos indígenas não se limitam a ações pontuais, mas passam a constituir estratégias de saúde global que dialogam diretamente com os ODS, sobretudo os relacionados à redução de desigualdades, à educação e à promoção da saúde.

A presente reflexão se limita a discutir a introdução de ideias da linguística e da semiótica entre enfermeiros e enfermeiras indígenas e não indígenas, sendo necessário ampliar a discussão para as áreas dos demais profissionais que atuam no SUS, observando-se as hierarquizações em saúde. Os povos indígenas enfrentam barreiras no acesso a serviços de saúde culturalmente adequados dentro e fora dos seus territórios, mas a interculturalidade permite trocas de saberes entre pajés, curandeiras, benzedeiras e pateiras, cujas tradições de cura podem ser eficazes a todos os usuários dos territórios.

Aponta-se, pois, a necessidade de executar pesquisas sobre as qualificações docentes, quanto ao acompanhamento das aprendizagens de discentes indígenas e não indígenas, sobre as dificuldades enfrentadas por estudantes de enfermagem indígenas na permanência em seus cursos universitários e sobre o desenvolvimento de tecnologias assistenciais com avaliação da semiótica, observando-se as diferentes etnias sob cuidado do SUS. Igualmente, impõem-se as demandas por estudos sobre intérpretes-tradutores indígenas no âmbito do SUS e sobre a construção e o uso de tecnologias, a exemplo de aplicativos e de dicionários, para facilitar a interpretação e a tradução dos diálogos com indígenas atendidos no âmbito do sistema público de saúde, objetivando a constituição de diagnósticos eficazes que ajudem os profissionais a realizar atendimentos mais eficientes.

Por outro lado, é oportuno agregar professores pesquisadores a espaços de diálogo sobre o tema, como grupos de trabalho específicos de órgãos públicos, para que participem diretamente da construção de políticas que fomentem discussões no âmbito da formação. Isso pode colaborar para o desenvolvimento e para a inserção de instrumentos inclusivos nos processos formativos, a fim de ampliar a discussão nos espaços de poder das universidades, como as pós-graduações, que concentram a criação de conhecimentos eurocêntricos.

## Data Availability

O conjunto de dados que dá suporte a este artigo não está disponível publicamente.

# Translation: Lingüística y semiótica en la formación y desempeño de personas indígenas y no indígenas en enfermeira

**DOI:** 10.1590/1980-220X-REEUSP-2025-0023es

**Authors:** Nádile Juliane Costa de Castro, Purupramare Lima Gavião, Antônio Luis Parlandin dos Santos, Fernanda Teixeira Paes

**Affiliations:** 1 Universidade Federal do Pará, Programa de Pós-Graduação em Enfermagem, Belém, PA, Brasil.; 2 Ministério da Saúde, Secretaria de Saúde Indígena, Distrito Guamá. Tocantins, Belém, PA, Brasil.; 3 Universidade Federal do Pará, Faculdade de Letras. Belém, PA, Brasil.

**Keywords:** Aprendizaje, Enfermeras y Enfermeros, Pueblos Indígenas, Desarrollo de Personal, Diversidad, Equidad e Inclusión

## Abstract

**Objetivo:**

Reflexionar sobre las contribuciones de la lingüística y la semiótica a la formación intercultural y al trabajo de enfermeros y enfermeras indígenas y no indígenas en el Sistema Único de Salud, destacando los desafíos, las potencialidades y las estrategias para la comunicación inclusiva en la atención a la salud de los pueblos indígenas.

**Método:**

Estudio teórico-reflexivo, basado en la literatura contemporánea, así como en la experiencia e identidad de sus autores, para dilucidar las contribuciones de estas ciencias, desde la perspectiva de la interculturalidad crítica.

**Resultados:**

Organizados en dos núcleos, siguiendo la secuencia de ideas sobre formación, implementación y acción: Lingüística y semiótica en la formación intercultural: estrategias para la equidad en la práctica profesional; y Traducción e interpretación de lenguas: caminos hacia la equidad en la atención de salud.

**Consideraciones finales:**

La inclusión de contenidos lingüísticos y semióticos específicos en los cursos de pregrado, asociada al desarrollo de herramientas bilingües y a la adopción de políticas respetuosas de las diversas cosmovisiones indígenas, refuerza los compromisos con la equidad y la justicia social en los servicios de salud prestados por el sistema público de salud brasileño.

## INTRODUCCIÓN

La implementación de políticas de salud y educación implica procesos inclusivos^(1)^, como el diálogo entre la diversidad lingüística y cultural en los espacios educativos y en los servicios de salud^(2)^. Estos procesos se aproximan a las discusiones sobre interculturalidad, aportando puntos teóricos y prácticos a la formación y al trabajo en salud de enfermeras indígenas y no indígenas. Por lo tanto, esto está en consonancia con entornos de aprendizaje sostenibles, inclusivos y equitativos^(1,3)^, basados en los principios del Sistema Único de Salud (SUS)^(4,5)^ y los Objetivos de Desarrollo Sostenible (ODS), especialmente los ODS 3 y 4 (Salud y bienestar y Educación de calidad), respectivamente, dando a las instituciones educativas acciones críticas, reflexivas y ciudadanas.

Este movimiento contribuye a ampliar el cuerpo de conocimientos y promueve rupturas con las epistemologías eurocéntricas, paradigma central en los actuales cursos de formación, convergiendo hacia la inclusión de epistemologías del Sur Global alineadas con la medicina indígena y las prácticas de cuidado de los 305 pueblos indígenas presentes en el territorio brasileño^(3)^. Esos cambios también proporcionan soporte teórico para problematizar las peculiaridades étnicas y las necesidades en salud de los territorios^(6)^, dentro de las perspectivas de los conceptos en salud y educación intercultural^(4,5)^. Además, contribuyen al abordaje de temas emergentes por sus movimientos multi-epistémicos, capaces de captar nuevos matices pedagógicos y dialogar con los habitualmente aplicados.

Dentro del SUS, ese escenario plural se identifica principalmente en el Subsistema de Atención a la Salud Indígena (SASISUS), que orienta la atención a la salud de los pueblos indígenas^(7)^, lo que exige un abordaje político que trate de la inclusión y de la interdisciplinariedad^(2,3)^. En este sentido, las ciencias del lenguaje pueden contribuir, a través de la lingüística y de la semiótica^(8)^, para proporcionar conocimientos sobre las formas de comunicación de los grupos étnicos atendidos por el SASISUS, con el objetivo de comprender los símbolos agregados e identificar los significados atribuidos a los elementos de las culturas en cuestión^(9)^.

La Enfermería, como protagonista en este escenario, exige avances en la diversidad y en la comunicación, importantes para actuar con seguridad y para las buenas prácticas en la asistencia sanitaria^(4)^, que consecuentemente no resulten en diagnósticos inexactos, tratamientos inadecuados y resultados desfavorables para la salud^(7)^. Por lo tanto, este estudio se justifica por sus propuestas de colmar las lagunas de conocimiento sobre la subutilización de las ciencias del lenguaje en la comunicación con los pueblos indígenas y dar respuesta a la Política Nacional de Atención a la Salud de los Pueblos Indígenas.

Además, el presente estado de la cuestión es fundamental en su alineación epistemológica con los autores indígenas, que reconocen las pluralidades étnicas y culturales y las cosmovisiones de sus sociedades. Esta discusión también demuestra la inseparabilidad entre la formación para trabajar en territorios indígenas y otras áreas del conocimiento, como la lingüística, que media la comunicación entre los usuarios y los Equipos Multidisciplinarios de Salud Indígena (EMSI), de la Atención Primaria de Salud (APS)^(7^), que incluye enfermeros, médicos, agentes indígenas de salud, entre otros.

Estudios en esta dirección han destacado los desafíos para la comunicación entre los EMSI y los pacientes indígenas; desafíos que ocurren dentro y fuera de los territorios, en la APS y en otros niveles de atención de salud, asociados sobre todo a la formación de pregrado. Por otro lado, estas oportunidades pueden hacer excelentes aportes, debido al acceso de los indígenas a la educación superior, a partir de la Ley 14.723/2023 (Ley de Cupos), que posibilita la formación de enfermeros indígenas.

Sin embargo, considerando las relaciones entre estudiantes indígenas y no indígenas, este acceso debe contar con apoyo pedagógico, interpretaciones lingüísticas y semióticas y comunicaciones inclusivas, aprehensiones sistémicas que aún deben ser exploradas^(7,10)^. Este aspecto pone en evidencia otras cuestiones de formación, como la superación de la intolerancia y del prejuicio lingüístico, que exige cambios epistemológicos para posibilitar intervenciones en la práctica social en salud, con el objetivo de lograr una atención diferenciada^(11,12,13)^.

Estas intervenciones promueven la equidad en la mediación de los servicios de salud, moldean nuevas estructuras e infraestructuras^(4)^ dentro de los servicios de salud, e impulsan investigaciones que enriquecen la comprensión de las necesidades de formación para trabajar en el SASISUS^(4,11)^, contribuyendo a reducir las inequidades sociales y de salud, con miras a promover cualificaciones inclusivas y socialmente comprometidas^(1)^.

Teniendo en cuenta lo anterior, se plantearon interrogantes sobre las mejores estrategias a utilizar para cualificar los procesos de aprendizaje en la formación de enfermeras indígenas y no indígenas, así como en su trabajo con usuarios indígenas, con el objetivo de señalar limitaciones y posibilidades para implementar procesos de aprendizaje y cuidado. A esto se suman los constructos que pueden desarrollarse de manera transversal, a través de la extensión y la investigación, y las estrategias de intervención que pueden/deben emprenderse para promover prácticas efectivas en salud para los pueblos indígenas. Finalmente, este estudio pretende reflexionar sobre las contribuciones de la lingüística y de la semiótica a la formación intercultural y al trabajo de las enfermeras indígenas en el SUS, destacando los desafíos, potencialidades y estrategias para una comunicación inclusiva en salud para los pueblos indígenas.

La lingüística y la semiótica proporcionan herramientas esenciales para la comprensión de los procesos de comunicación, especialmente en contextos interculturales y multilingües, como la formación de enfermeros que trabajan con pueblos indígenas. Estos campos permiten aproximaciones críticas y reflexivas a las formas en que el lenguaje y los símbolos configuran las relaciones en salud, contribuyendo directamente a la formación de profesionales culturalmente competentes. En este estudio, las reflexiones se basarán en un diálogo con autores del Sur Global, observando los estudios sobre salud intercultural, que han guiado los debates sobre la atención sanitaria indígena, con el fin de permitir el diálogo entre las identidades de los autores indígenas y la formación académica.

### Aspectos Conceptuales de la Lingüística y la Semiótica y Perspectivas Indígenas

La lingüística es la ciencia que estudia el lenguaje verbal en sus múltiples dimensiones, abarcando aspectos descriptivos, históricos y teóricos del lenguaje como sistema estructurado de comunicación y su constante elaboración y reelaboración^(8)^. Se diferencia de la semiótica, dedicada al estudio de los signos, que abarca los sistemas y procesos representados por símbolos y signos^(14)^, en sus formas, tipos y efectos. Mientras que la lingüística se centra en las estructuras y el funcionamiento de las lenguas en sus dimensiones deíctico-referencial, lógica, pragmático-discursiva, sintáctica y semántica^(15)^, la semiótica investiga cómo se construyen, comparten e interpretan los significados a través de los diferentes sistemas de signos presentes en las distintas culturas^(14)^.

En este sentido, reconocer las subáreas de la lingüística, como la semántica, la pragmática y la sociolingüística, permite comprender los significados, los usos del lenguaje en determinados contextos y las relaciones sociales, respectivamente^(8)^. Además, distinguir el papel de la semiótica cultural es fundamental para el estudio de los signos en contextos indígenas, debido a la multimodalidad (verbal, visual, gestual, espacial) identificada en ellos y a la incorporación de diferentes elementos en el análisis de las prácticas simbólicas de diferentes grupos étnicos^(16,17)^. Esta separación es fundamental para comprender cómo los campos de conocimiento contribuyen de manera diferente a la formación intercultural en salud, especialmente en contextos multilingües^(9)^.

Por otro lado, es importante destacar que la lingüística y la semiótica tienen sus bases conceptuales y metodológicas construidas sobre epistemologías occidentales^(8,14)^. En este sentido, existen riesgos en aplicar los conceptos eurocéntricos que las caracterizan a las realidades indígenas sin la debida problematización, ya que los pueblos indígenas del territorio brasileño tienen sus propios sistemas de producción e interpretación de significados, intrínsecamente ligados a sus cosmovisiones^(16)^. Por lo tanto, es necesario observar una «semiótica indígena» que reconozca sus propios sistemas de signos y significados, que se manifiestan a través de pinturas corporales, gráficos, cantos, narraciones, rituales y sus propias relaciones con el territorio^(17)^.

## MÉTODO

Se trata de un estudio teórico-reflexivo, basado en la literatura científica sobre lingüística y semiótica, relativo a la formación de enfermeros indígenas y no indígenas, así como a las percepciones, experiencias e identidades de sus autores, con el fin de dilucidar las relaciones que se establecen entre la formación y el trabajo de estos profesionales. Esta investigación se realizó entre noviembre de 2023 y diciembre de 2024 y se basó en la denominada educación intercultural de Gersen Baniwa^(16)^, a través de la cual la autora plantea que es necesaria una mirada crítica a las epistemologías que se han aplicado en los procesos de formación, y que es imprescindible romper con esta hegemonía y dialogar con otras formas de pensar.

Las cualificaciones lingüísticas y culturales son centrales y subsidian mejoras en la eficacia comunicacional de la interacción entre enfermeros y pacientes indígenas^(18)^, aspecto que, a la luz del pensamiento y del lenguaje, trasciende la comprensión de los fenómenos sociales. En la formación de estos trabajadores, tales mejoras tienen el potencial de desarrollar acciones interculturales complejas^(2)^, tanto a nivel social en un primer momento- como a nivel individual en una etapa posterior^(14)^ , decisivas para comprender y mejorar la comunicación y el aprendizaje en contextos multiculturales y multilingües en salud^(8,9)^.

Para que la formación de enfermeros sea inclusiva, se defiende que esté mediada por conceptos indigenistas de la lingüística y de la semiótica^(17)^, lo que debería contribuir a la promoción de prácticas comunicativas más integrales, a la reducción de los desafíos lingüísticos y culturales dentro del SUS y al fortalecimiento de políticas públicas orientadas a la equidad. Este paradigma también puede subsidiar un aprendizaje más holístico en la enfermería^(19)^ y los objetivos de los ODS 3 y 4, en particular el desarrollo y la formación de profesionales de la salud (punto 3c) y la valoración de la diversidad cultural (punto 4.7)^(19,20)^.

Este texto se estructura en torno a dos núcleos temáticos: Lingüística y semiótica en la formación intercultural: estrategias para la equidad en la práctica profesional; y Traducción e interpretación de lenguas: caminos para la equidad en salud. Se trata de un trabajo teórico-reflexivo y no necesita ser evaluado por el sistema CEP/CONEP.

### Desarrollo

Como punto de partida, es necesario entender la lengua como instrumento técnico y como medio de construcción de relaciones interculturales, mediadas por símbolos, valores y contextos específicos de las comunidades indígenas. En este sentido, a partir de las experiencias de los autores, es posible identificar desafíos y potencialidades para identificar estrategias pragmáticas.

A partir del diálogo propuesto, se entiende que la interacción social es central para el desarrollo del pensamiento y del lenguaje^(16)^, reflejándose directamente en la formación de los profesionales de la salud. Por otro lado, la cuestión involucra otros aspectos, como la identificación de la diversidad étnica^(16)^, los territorios indígenas y los establecimientos de salud y sus tipologías (Figura 1), para que los profesionales indígenas y no indígenas reciban una formación que valorice sus lenguas maternas y comprenda los significados simbólicos atribuidos a la salud y a la enfermedad por cada grupo – y sus implicaciones en los procesos de atención.

**Figura 1:** Representación del diálogo en la formación intercultural de enfermería.

Fuente: autores, 2025.

### Lingüística y Semiótica en la Formación Intercultural: Estrategias para la Equidad en la Práctica Profesional

La competencia intercultural^(10,15)^ y el reconocimiento del multilingüismo son cruciales para las enfermeras, especialmente para las que trabajan en el SASISUS en regiones con diferentes pueblos indígenas. Estos aspectos son fundamentales para la obtención de una atención sanitaria equitativa^(4,5)^, en la que la capacidad de identificar y desenvolverse en varios idiomas amplía la comprensión de los matices culturales^(4)^, que son intrínsecos a la comunicación, y la interpretación de los mensajes en salud^(13)^. Además, establecer una comunicación culturalmente sensible enriquece la interacción entre el paciente y la enfermera^(5)^ y también promueve un enfoque más holístico de la atención^(13)^.

La mejora de la comunicación abarca la capacidad de comprender y hacerse comprender en los espectros multilingüe^(18)^y multicultural que son intrínsecos a la eficacia y la calidad de la atención prestada en el SUS, que trasciende la simple transmisión de información. Del mismo modo, esta mejora surge como un pilar fundamental para valorar la diversidad cultural, reconociendo que cada individuo trae consigo conocimientos y prácticas que conforman su percepción de la salud, como se señala en los ODS^(19,20)^. Cuando está bien estructurada, esta mejora puede equipar a las enfermeras para transferir adecuadamente conocimientos sanitarios críticos^(5)^, construir relaciones de confianza y empatía, y sistematizar correctamente los cuidados necesarios para la denominada atención humanizada, especialmente en el SASISUS^(10,13)^.

Además, la capacidad de comunicar y comprender los matices culturales amplía la accesibilidad y la inclusión en la atención sanitaria^(2,13)^, facilitando la aplicación de estrategias de promoción de la salud y prevención de la enfermedad que respeten las singularidades de cada grupo étnico^(13)^. Así, en la interfaz entre lingüística, semiótica y equidad^(8,9)^, la inclusión de indígenas en diferentes niveles de educación, como pregrado y posgrado^(1,3)^, es un puente para el desarrollo profesional^(19,20)^ y el fortalecimiento de la representatividad cultural y epistémica en los espacios educativos.

Además, esa mejora puede promover la construcción de prácticas de atención más humanizadas y respetuosas, alineadas con las especificidades y cosmovisiones indígenas, así como contribuir a la reducción de las inequidades en salud y a la consolidación de una formación crítica y socialmente justa en el SUS. Para ello, es necesario insertar estos paradigmas en los procesos educativos y reconocer el papel fundamental de la lingüística en la conducción del proceso, ya que se basa en recursos que analizan sus relaciones estructurales^(5)^, abarcando las dimensiones semántica, deíctico-referencial y pragmático-discursiva^(8,9,15)^. Este enfoque tiene en cuenta las diversidades y complejidades que rigen los diferentes contextos comunicativos, incluyendo los procesos históricos de formación de la lengua y también las interpretaciones de los signos y símbolos culturales, presentes en los principios de la semiótica^(8)^.

En la práctica, esto significa identificar y comprender las diversas lenguas asociadas a identidades étnicas que han ingresado en las universidades públicas brasileñas, a menudo como parte de un proceso de reivindicación territorial^(3,10,21)^. Estos grupos étnicos, vinculados a territorios indígenas, pasan a formar parte de las Instituciones de Educación Superior (IES), trayendo consigo una diversidad de cosmovisiones^(3)^. A nivel semántico, estas expresiones se manifiestan de diversas maneras: en el modelo deíctico-referencial, demandan estudios sobre los contextos e historias de estos grupos; y en el ámbito pragmático, reflejan cuerpos indígenas.

Esta pragmática de los cuerpos indígenas se refiere a la forma en que comunican y expresan significados, a través de gestos, posturas e interacciones culturales específicas, que varían según los contextos socioculturales e históricos. Además, existen percepciones semántico-fonéticas, que engloban las diferentes interpretaciones de los pueblos sobre los significados de las palabras (semántica) y los sonidos de las lenguas (fonética), que revelan aspectos relacionales de sus acciones en el mundo^(8,9)^.

Por lo tanto, la pragmática de los cuerpos indígenas implica gestos, posturas e interacciones culturales específicas que son cruciales para la relación enfermera-paciente en contextos multiculturales. Sin embargo, esas formas de expresión suelen ser ignoradas por los currículos de formación basados en epistemologías coloniales, que refuerzan las centralidades del portugués y los patrones de pensamiento del Norte Global. Como resultado, muchos profesionales no indígenas desconocen códigos culturales que podrían cualificar y mejorar la atención, perpetuando las barreras de comunicación en relación con los usuarios, sin excluir las prácticas de salud indígenas^(3,17,22)^.

En este contexto, es necesario observar las cerca de 160 lenguas indígenas y dialectos hablados por los 305 grupos étnicos presentes en el territorio brasileño, que se organizan en dos troncos lingüísticos – Tupi y Macro-Jê – que comprenden familias y subgrupos, con sus peculiaridades y diversidades, dependiendo de los pueblos indígenas que las utilizan. La lengua Tupi se concentra en la mayor parte de los territorios brasileños y está formada por diez familias lingüísticas, mientras que la Macro-Jê tiene nueve familias y está muy concentrada en la zona de la Amazonia Legal, pero no sólo allí. Además, existen otras familias lingüísticas aisladas, con un total de 20 lenguas: aikaná; arawá; arúak; guaikuru; iranxe; jabuti; kanoé; karib; katuxina; koazá; máku; makú/mura; nanambikwára; pano; trumái; tikúna; tukano; txapakúra; y yanomami^(22)^.

Sin embargo, la falta de reconocimiento de esta pluralidad lingüística es consecuencia directa del uso de un modelo colonial que favoreció la lengua portuguesa y menoscabó las lenguas originarias. Es necesario revisar ese pensamiento, con el objetivo de establecer la atención en la lengua materna de las personas atendidas, para que los usuarios indígenas sean bien recibidos y puedan expresarse con claridad, de modo que el profesional de salud tenga éxito en la prescripción de exámenes y/o medicamentos.

Dado que es a través del lenguaje que se da la comunicación, cuando se habla de pueblos indígenas, es necesario comprenderlos, a partir de sus historias sociales y troncos lingüísticos, en el contexto del SASISUS y de la salud intercultural^(2,15)^, observando cómo las percepciones semántico-fonéticas de la multiplicidad etnolingüística^(18)^ impactan en los procesos de atención a la salud. Esto entra en conflicto con el hecho de que sólo la lengua portuguesa sea utilizada para mediar procesos en el SUS, debido a la realidad del SASISUS, que incorpora diferentes etnias en su estructura administrativa – los Distritos Especiales de Salud Indígena (DSEI) – que no se evidencian en los procesos de trabajo del SUS, que son generalistas y de sesgo biomédico^(11)^.

En este sentido, el predominio de la lengua portuguesa como mediadora en los procedimientos del SUS desconoce la diversidad lingüística de los pueblos indígenas y crea barreras significativas para la atención sanitaria de estos grupos, ya que la ausencia de traductores o materiales bilingües dificulta la transmisión de información crítica en muchos escenarios dentro del SASISUS. Este matiz pone de manifiesto una deficiencia crítica en la formación: la falta de enfoques técnicos y pedagógicos adecuados para atender a los pueblos indígenas, debido a déficits de competencias, lo que se traduce en una atención que no respeta las especificidades culturales y lingüísticas. Para superar estas limitaciones, es fundamental implementar políticas lingüísticas inclusivas que garanticen la producción de materiales bilingües, la formación de intérpretes culturales y la capacitación de los equipos de salud para lidiar con las pluralidades lingüísticas y culturales presentes en los contextos del SUS.

En esta línea, se reflexiona sobre los diferentes contextos culturales que pueden ser identificados al realizar abordajes semióticos en enfermería, ya que los signos verbales representan ideas diferentes de acuerdo con los diversos troncos lingüísticos actualmente en uso^(22)^. Por lo tanto, es de interés incluir, discutir y comprender los elementos simbólicos^(3)^ presentes en las convergencias y divergencias que pueden ser identificadas en los espacios de vida de los pueblos indígenas por los equipos de enfermería, coincidentes o no con los territorios en los que actúan, en particular aquellos que están directamente relacionados con los rituales que influyen en la adhesión a los cuidados médicos del SUS^(13)^.

En la práctica asistencial, ese pragmatismo se refleja en gestos y silencios, portadores de significados culturales. Por ejemplo, el silencio puede indicar consentimiento en lugar de desinterés. Las percepciones semántico-fonéticas surgen cuando la traducción de términos sanitarios técnicos, como «virus», adquiere significados diferentes en las lenguas indígenas, lo que afecta a la comprensión del diagnóstico y a la aceptación del tratamiento. Esto revela los problemas y escenarios que enfrentan los estudiantes indígenas cuando ingresan a la educación superior.

La hegemonía eurocéntrica de las IES resulta particularmente desafiante y opresiva para estos estudiantes, quienes enfrentan retos lingüísticos y epistemológicos al enfrentarse a un sistema educativo que rara vez reconoce o valora sus conocimientos y formas de entender el mundo. Reconocer estas condiciones es esencial para una comunicación que trascienda los conocimientos técnicos y sea capaz de contextualizar los cuidados. Es en este campo donde las aportaciones de la lingüística y la semiótica surgen como respuestas fundamentales, ya que ofrecen herramientas teóricas y metodológicas que permiten mejorar los entornos formativos y educativos.

Para que la formación intercultural sea una realidad, es fundamental que los programas de pregrado de enfermería incluyan módulos específicos de lingüística y semiótica, para desarrollar competencias comunicativas que tengan en cuenta las pluralidades culturales y lingüísticas de los pueblos indígenas. Estas asignaturas pueden incluir tanto aspectos teóricos – introduciendo conceptos de análisis semántico, pragmática y semiótica – como prácticas simuladas de cuidados, que permitan a los estudiantes practicar anamnesis y entrevistas de salud en contextos multiculturales, apoyando una educación de calidad^(19,20)^. De esta forma, al egresar, los estudiantes podrán promover acciones inclusivas al entrar en contacto con las particularidades de la atención en salud de los pueblos indígenas.

Por otro lado, los estudios sobre lenguas indígenas han sido atravesados por cuestiones estructurales^(9)^, discursos sobre intolerancia y prejuicios en los procesos de aprendizaje^(10)^, aspectos que señalan la necesidad de diálogo sobre este tema en la titulación de enfermeros indígenas y no indígenas. Al abordar el tema, se pueden mencionar algunos puntos para orientar el debate: los discursos sobre prejuicios, intolerancia y variedad lingüística; la producción de herramientas no inclusivas; la enseñanza como sujeto de construcciones sociales; y el papel de los espacios educativos en la inclusión social^(3,12)^.

En esta línea, se debe enfatizar la implementación de propuestas transversales que aborden los fenómenos observados en diferentes lenguas, traducidos en tecnologías de atención, gestión y educación, que tengan potencial para ser utilizadas dentro del SUS. Estas tecnologías median los procesos de atención en salud y educación, desde la formación hasta el desarrollo de competencias, a través de prácticas que buscan productos inclusivos, identitarios y diversos^(21)^, como los diccionarios, para mediar la atención prestada por los equipos de enfermería en el DSEI.

La conexión entre la lingüística y las tecnologías del cuidado va más allá de la simple traducción de palabras; estas herramientas permiten a los profesionales de la salud adaptar sus comunicaciones a las especificidades culturales y lingüísticas de las comunidades indígenas, ayudando a explicar diagnósticos y procedimientos, y a componer guías más accesibles. Esta adaptación puede llevar las descripciones de conceptos a las lenguas indígenas, evitando malentendidos, puede promover una comunicación más eficaz y puede contribuir a reforzar la identidad cultural y la reintegración simbólica y práctica de los territorios indígenas en el marco del SASISUS.

A través de este enfoque, es posible realizar capacitaciones estructuradas sobre la inclusión y el diálogo de la lingüística y la semiótica en los proyectos y herramientas pedagógicas, tanto para la transmisión adecuada de la información como para la construcción de relaciones de confianza y empatía en los procesos de atención^(13,21)^. Para ello, es necesario valorar la individualidad y promover relaciones de respeto y comprensión mutua, actualizando y mejorando las competencias comunicativas de los profesionales^(13)^, desde el momento en que los estudiantes inician sus estudios de grado.

Por lo tanto, la inclusión de la lingüística y la semiótica en la formación de enfermería favorece las mediaciones necesarias dentro del SUS, para que las experiencias en servicios de salud sean positivas. Por lo tanto, el proceso debe terminar con la sensibilización de la gestión educativa, del personal docente y de los estudiantes indígenas y no indígenas a esta realidad. Esta es una oportunidad única para implementar estudios de semiótica en el desarrollo de productos^(21)^, por ejemplo.

En la formación de enfermeros, esas discusiones convergen hacia un objetivo común: promover la equidad y la inclusión en salud cuando se trabaja en los servicios del SUS. La formación de profesionales sensibles a las especificidades culturales y lingüísticas de los pueblos indígenas es esencial para superar las limitaciones de un sistema históricamente basado en epistemologías coloniales, que se observan en toda la formación académica^(23)^. Al mismo tiempo, ese cambio en las formas de actuación se refleja en movimientos de reintegración simbólica y práctica de los territorios, reafirmando la centralidad de las cosmovisiones indígenas en la construcción de ejercicios y en la producción de tecnologías de salud inclusivas^(24)^. Así, la articulación entre las dimensiones de salud y educación^(24,25)^ es un paso significativo para transformar el SUS en un sistema verdaderamente intercultural, alineado con las necesidades de los pueblos indígenas y los principios de la justicia social.

En la interfaz con la equidad, el movimiento en torno al tema no sólo alcanza objetivos globales^(1)^, especialmente los relacionados con la salud y la educación^(^25), sino que también constituye un instrumento político que afirma la restitución de los territorios^(3)^. A escala de la salud mundial, esto representa la igualdad de derechos, las diferencias y la diversidad, e incluso ofrece oportunidades para reducir las desigualdades sociales enumeradas en los distintos ODS^(1)^.

### Traducción e Interpretación de Lenguas: Vías para la Equidad en Salud

La traducción y la interpretación lingüísticas desempeñan un papel fundamental en la promoción de la equidad en el SUS, así como en la superación de las barreras lingüísticas en los servicios de salud^(4,5)^. Por otro lado, cuando no se superan, estos obstáculos ponen en peligro los diagnósticos, la adherencia al tratamiento y los resultados en salud, reforzando las desigualdades estructurales. En este sentido, la inclusión de intérpretes- traductores indígenas es necesaria para mediar la comunicación y respetar las cosmovisiones y valores culturales de los pueblos indígenas, desde las perspectivas de la interculturalidad, el multilingüismo^(18)^ y la instrumentalización, a través de la extensión y la investigación^(26)^.

Asimismo, la traducción y la interpretación son cruciales para construir relaciones de confianza, proporcionando una cultura de comunicación inclusiva que garantice la seguridad del paciente^(5)^. Por otro lado, la falta de traductores-intérpretes y de políticas públicas eficaces en este sentido dificulta el acceso a los derechos fundamentales, que confieren dignidad humana a las personas. Por lo tanto, es esencial garantizar la presencia de estos profesionales alfabetizados en las comunidades indígenas para alcanzar el principio de igualdad, en el que todos puedan entender y ser entendidos, un derecho avalado por la Constitución Federal y el Convenio 169 de la Organización Internacional del Trabajo^(26-28)^.

Por lo tanto, hay dos puntos a destacar: la inclusión de diálogos lingüísticos en la formación de enfermeros; y la contratación de intérpretes-traductores para mediar en las comunicaciones en el SUS. El primero amplía la mirada crítica y reflexiva, ya que las discusiones y los diálogos sobre la diversidad y las cosmovisiones indígenas tienen un papel sociopolítico^(25)^, y el segundo destaca el papel y el lugar de las lenguas^(25)^, ya que las aproxima a los valores simbólicos de los pueblos indígenas, poniendo en práctica el carácter político observado en las diferentes formas de comunicarse^(8,9,25)^. De esta manera, se hace evidente la función social de insertar paradigmas lingüísticos y semióticos en estos contextos, ya que pueden construir caminos hacia la equidad^(27)^.

Por eso, no basta con promover discusiones específicas para valorizar la lingüística y la semiótica; es necesario que esas ciencias ocupen un lugar y una función^(25)^. Esto se puede lograr integrándolas al currículo, es decir, ofreciendo programas de formación y certificación que generen contratos de trabajo en APS, hospitales y clínicas^(4,5)^, especialmente para quienes conocen las lenguas indígenas además de las enfermeras-, ya que involucra no sólo aspectos formativos, sino también interaccionales y culturales, entre otros^(25)^. Esta lógica está en consonancia con la eficacia y la resolución, así como la relación con el otro, que beneficia a los individuos y ratifica el compromiso político de la enfermería^(26,27)^.

La presencia de intérpretes ya está prevista en los procesos judiciales, a petición de la Fundación Nacional del Indígena (FUNAI), lo que está en consonancia con los derechos internacionales y humanos. En el caso de la salud, hay pocos registros de ello en las acciones de la FUNAI y del SASISUS, considerando que el apoyo de intérpretes indígenas puede ser utilizado para ayudar en la atención prestada a los indígenas cuando necesitan salir de las aldeas.

Además, ese cambio de mentalidad es fundamental para mediar en los cuidados prestados por EMSI, formada por enfermeros indígenas que dan instrucciones sobre cuidados humanizados y gestión de la salud^(26)^. Sin embargo, hay que tener en cuenta algunos puntos, como la integración de estos trabajadores en la rutina diaria de EMSI, con sus horarios y turnos, para garantizar su disponibilidad. La participación del intérprete en todas las etapas de la atención (desde el cribado hasta el alta) profundiza la comprensión de las dimensiones culturales y lingüísticas que afectan al diagnóstico y al tratamiento, ya que, además de traducir términos técnicos, actúan como mediadores socioculturales, promoviendo la práctica intercultural.

En contextos clínicos, la presencia de intérpretes y traductores garantiza una comunicación eficaz que trasciende la simple traducción lingüística, ya que abarca la interpretación de contextos, valores y prácticas culturales específicas^(25)^, como se señala en los objetivos globales^(19,20)^. Los diagnósticos inexactos y los tratamientos inadecuados pueden surgir de malentendidos derivados de la ausencia de puentes interpretativos^(^26), que no sólo traducen palabras, sino también matices culturales y conceptos de salud y enfermedad, que son intrínsecos a las cosmovisiones indígenas^(29)^.

Además, los pueblos indígenas tienen una relación intrincada y profunda con su entorno, por lo que es esencial interpretar adecuadamente estas conexiones. Por ejemplo, las variaciones en la temperatura de los ríos no son sólo acontecimientos ecológicos para estas comunidades, sino que pueden tener implicaciones directas para su salud y bienestar, ya que pueden influir en la salud de los ecosistemas acuáticos, repercutir en la dieta y la medicina indígenas e incluso en las prácticas culturales de estos pueblos. En este caso, el papel de un intérprete va más allá de la traducción: facilitan la comprensión interdisciplinar de las complejas interacciones entre cultura, salud y medio ambiente^(30)^.

Mientras que los traductores se ocupan principalmente de material escrito, los intérpretes pueden actuar de forma oral e inmediata durante las consultas y los procedimientos de enfermería. En este sentido, establecer protocolos de trabajo que definan claramente las responsabilidades de cada profesional en el campo de la comunicación en salud es un punto de partida para evitar el solapamiento de las comunicaciones.

La mediación de intérpretes que acompañan a los indígenas en tratamiento en el SUS favorece la comunicación para su adherencia al tratamiento, así como la eficacia de los resultados, mientras que, debido a la organización de las familias indígenas, hay una nueva generación de indígenas bilingües que, en general, acompañan a estos usuarios. Sin embargo, existe una demanda reprimida, que favorece la promoción de estos profesionales más allá de las acciones como enfermeros indígenas, ya que los no indígenas pueden producir comunicaciones dispares y genéricas, debido a su desconocimiento de las culturas que los caracterizan.

Esto se debe a que la organización lingüística de cada grupo étnico es única y representa un contexto cultural particular, lo que didácticamente indica que el trabajo de los intérpretes-traductores indígenas puede resultar en una mejor comprensión de las necesidades inherentes a ese grupo étnico que la interlocución realizada por el personal no indígena, que puede presentar interpretaciones genéricas^(30)^. Dentro del SUS, la incorporación de estos intérpretes puede ser esencial en áreas como la limpieza, la seguridad y las enfermedades, en las que la terminología y las prácticas específicas de un grupo étnico pueden malinterpretarse o simplificarse en exceso cuando las traducen personas no indígenas. Al aplicar una traducción culturalmente competente, el SUS puede garantizar una prestación de servicios más eficaz y adaptada.

Para el SUS, esto significa el acceso a la información a través de la lengua materna, que reconoce e incluye el multilingüismo. Ante la exigencia de acciones equitativas, una traducción eficaz hace posible la comunicación. Por lo tanto, es importante reconocer las diferentes simbologías que las lenguas indígenas presentan en cada comunidad, o no, porque algunos signos no se pueden traducir. En cualquier caso, esa reflexión es capaz de promover una mejor salud, así como implementar acciones previstas en las conferencias de salud, que reconocen la necesidad de traducción e interpretación en las redes de atención^(11)^.

Para sensibilizar a los EMSI, es necesaria la formación continua, es decir, que los profesionales sanitarios puedan recibir formación permanente sobre el trabajo como intérpretes y sobre cómo respetar y atender las necesidades lingüísticas de los pacientes. También es posible ofrecer políticas de inclusión lingüística, que pueden aplicarse para garantizar el respeto de los derechos lingüísticos de los pacientes^(25)^.

En este contexto, se destaca la importancia fundamental de las enfermeras y los estudiantes indígenas como mediadores culturales y lingüísticos capaces de promover una comunicación eficaz. Como se mencionó, estos profesionales enfrentan importantes desafíos durante su formación académica, especialmente los relacionados con el lenguaje técnico de la salud, lo que representa un obstáculo adicional para aquellos que no tienen el portugués como lengua materna. Sin embargo, el aprendizaje de la lengua portuguesa y de la terminología técnica en salud puede llevarse a cabo mediante programas de tutoría, material didáctico adaptado y glosarios bilingües.

Deben preverse alianzas entre las instituciones de enseñanza superior y los líderes indígenas para garantizar procesos de certificación que reconozcan las competencias lingüísticas y culturales inherentes al trabajo. Además, los programas de formación continua para los equipos de enfermería y otros profesionales de la salud son esenciales, ya que refuerzan la comprensión de la importancia de la interculturalidad y el multilingüismo en la práctica clínica.

En este sentido, la colocación de intérpretes y traductores en el SUS, combinada con la sensibilización de los equipos de salud a las especificidades culturales de los pueblos indígenas, no sólo refuerza la equidad en la atención, sino que también avanza hacia los objetivos de los ODS^(19,20)^. La presencia de profesionales bilingües y culturalmente competentes también contribuye directamente a reducir las desigualdades (ODS 10) al facilitar el acceso de los pueblos indígenas a la información y a los servicios de salud, así como a promover una salud de calidad (ODS 3) al garantizar diagnósticos más precisos y tratamientos más acordes con las cosmovisiones locales. Así, la adopción de políticas y prácticas que reconozcan e incluyan las lenguas indígenas beneficia a las comunidades implicadas y refuerza los compromisos globales con la dignidad humana y la justicia social, basados en la educación inclusiva, tal y como se establece en el ODS 4^(19,20)^.

## CONSIDERACIONES FINALES

Este estudio trae contribuciones sobre la capacidad de comunicarse de forma inclusiva en el campo de la salud indígena, a partir de la lingüística y la semiótica, buscando comprender los matices culturales asociados a este modo específico de comunicación, en la medida en que este ejercicio amplía la accesibilidad y la inclusión de este público en el ámbito de la atención en salud del SUS. Evidentemente, esta investigación enriquece la producción de conocimiento al romper con las bases epistemológicas coloniales que caracterizan el proceso de comunicación aquí descrito.

La inclusión de contenidos lingüísticos y semióticos específicos en los programas de pregrado, junto con el desarrollo de herramientas bilingües y la adopción de políticas que respeten las diversas cosmovisiones indígenas, refuerza los compromisos con la equidad y la justicia social. En este escenario, la reintegración de los territorios y el reconocimiento de las lenguas y prácticas culturales de los pueblos indígenas no se limitan a acciones puntuales, sino que se convierten en estrategias globales de salud que dialogan directamente con los ODS, especialmente los relacionados con la reducción de las desigualdades, la educación y la promoción de la salud.

Esta reflexión se limita a discutir la introducción de ideas lingüísticas y semióticas entre enfermeros indígenas y no indígenas, siendo necesario ampliar la discusión a las áreas de otros profesionales que actúan en el SUS, observando las jerarquías en salud. Los pueblos indígenas enfrentan barreras para acceder a servicios de salud culturalmente apropiados dentro y fuera de sus territorios, pero la interculturalidad permite el intercambio de conocimientos entre chamanes, curanderos, benzedeiras y pateiras (curadoras), cuyas tradiciones curativas pueden ser efectivas para todos los usuarios de los territorios.

Por lo tanto, es necesario realizar investigaciones sobre la cualificación de los docentes, la forma de acompañar el aprendizaje de los estudiantes indígenas y no indígenas, las dificultades que enfrentan los estudiantes indígenas de enfermería para permanecer en sus cursos universitarios y el desarrollo de tecnologías de cuidado con una evaluación de la semiótica, observando los diferentes grupos étnicos bajo el cuidado del SUS. También se demandan estudios sobre intérpretes-traductores indígenas dentro del SUS y sobre la construcción y uso de tecnologías, como apps y diccionarios, para facilitar la interpretación y traducción de diálogos con indígenas atendidos dentro del sistema público de salud, con el objetivo de establecer diagnósticos eficaces que ayuden a los profesionales a prestar una atención más eficiente.

Por otro lado, sería una buena idea involucrar a los profesores investigadores en espacios de diálogo sobre el tema, como grupos de trabajo específicos dentro de los organismos públicos, para que puedan participar directamente en la construcción de políticas que fomenten discusiones en el ámbito de la formación. Esto podría ayudar a desarrollar e insertar herramientas inclusivas en los procesos de formación, con el fin de ampliar la discusión en los espacios universitarios de poder, como los cursos de posgrado, que concentran la creación de conocimiento eurocéntrico.

## Data Availability

El cunjunto de fatos que respalda este articulo no está disponible públicamente.
